# Fmrp Interacts with Adar and Regulates RNA Editing, Synaptic Density and Locomotor Activity in Zebrafish

**DOI:** 10.1371/journal.pgen.1005702

**Published:** 2015-12-04

**Authors:** Adi Shamay-Ramot, Khen Khermesh, Hagit T. Porath, Michal Barak, Yishay Pinto, Chaim Wachtel, Alona Zilberberg, Tali Lerer-Goldshtein, Sol Efroni, Erez Y. Levanon, Lior Appelbaum

**Affiliations:** 1 The Mina and Everard Goodman Faculty of Life Sciences, Bar-Ilan University, Ramat-Gan, Israel; 2 The Leslie and Susan Gonda Multidisciplinary Brain Research Center, Bar-Ilan University, Ramat-Gan, Israel; University of Michigan, UNITED STATES

## Abstract

Fragile X syndrome (FXS) is the most frequent inherited form of mental retardation. The cause for this X-linked disorder is the silencing of the fragile X mental retardation 1 (*fmr1*) gene and the absence of the fragile X mental retardation protein (Fmrp). The RNA-binding protein Fmrp represses protein translation, particularly in synapses. In *Drosophila*, Fmrp interacts with the adenosine deaminase acting on RNA (Adar) enzymes. Adar enzymes convert adenosine to inosine (A-to-I) and modify the sequence of RNA transcripts. Utilizing the *fmr1* zebrafish mutant (*fmr1*-/-), we studied Fmrp-dependent neuronal circuit formation, behavior, and Adar-mediated RNA editing. By combining behavior analyses and live imaging of single axons and synapses, we showed hyperlocomotor activity, as well as increased axonal branching and synaptic density, in *fmr1*-/- larvae. We identified thousands of clustered RNA editing sites in the zebrafish transcriptome and showed that Fmrp biochemically interacts with the Adar2a protein. The expression levels of the *adar* genes and Adar2 protein increased in *fmr1*-/- zebrafish. Microfluidic-based multiplex PCR coupled with deep sequencing showed a mild increase in A-to-I RNA editing levels in evolutionarily conserved neuronal and synaptic Adar-targets in *fmr1*-/- larvae. These findings suggest that loss of Fmrp results in increased Adar-mediated RNA editing activity on target-specific RNAs, which, in turn, might alter neuronal circuit formation and behavior in FXS.

## Author Summary

The most frequent inherited mental retardation disorder is fragile X syndrome, which is characterized by learning disabilities, cognitive impairment, anxiety, and hyperactive behavior. The genetic cause of this disorder is the silencing of the *fmr1* gene, which encodes the RNA-binding protein Fmrp. This protein inhibits the production of various proteins in the brain and interacts with the Adar enzyme, which converts the nucleotide A into I in RNAs. However, it is unclear by which mechanism the loss of Fmrp affects the sequence of neuronal genes and, ultimately, brain function. Here, we used the *fmr1* mutant zebrafish (*fmr1*-/-), which enables high-throughput genetics and live imaging experiments in a transparent and evolutionarily conserved brain. We found that loss of Fmrp altered neuronal circuit formation. Furthermore, similar to human patients, the *fmr1*-/- larvae were hyperactive. Biochemical assays showed that Fmrp interacts with the Adar2a protein, which is increased in *fmr1*-/- larvae. Thus, we characterized global RNA editing in the zebrafish transcriptome and used a microfluidic-based high-throughput technique to accurately quantify RNA editing levels. Loss of Fmrp resulted in a mild increase in RNA editing in the coding sequences of conserved synaptic genes. These findings propose that altered RNA editing levels may affect neuronal and behavioral deficiencies in FXS.

## Introduction

Fragile X syndrome (FXS) is the most common single-gene inherited neurodevelopmental disorder causing mental retardation. This disorder is characterized by an array of behavioral and cognitive disabilities, including autism, anxiety, epileptic seizures, hyperactivity, attention deficits, and mild craniofacial abnormalities [1,2]. The cause for FXS is a genetic loss of fragile X mental retardation protein (Fmrp) due to transcriptional silencing of the fragile X mental retardation 1 (*fmr1*) gene. In the 5'-untranslated region (5’-UTR) of *fmr1*, an expansion of more than 200 CGG trinucleotide repeats results in abnormal DNA hypermethylation and diminished mRNA expression [3]. Fmrp is predominately a cytoplasmic protein and consists of two ribonucleoprotein K homology (KH) domains and a GAR/RGG (glycine-arginine-rich) box. It is an RNA-binding protein that is essential for the function of the central nervous system [2–4] (CNS). In neurons, it regulates neurite transport of a subset of mRNAs and inhibits protein translation by blocking both initiation and elongation. In synapses, upon metabotropic glutamate receptor (mGluR) stimulation, the translation of Fmrp-targeted mRNAs is inhibited, allowing α-amino-3-hydroxy-5-methyl-4-isoxazolepropionic acid (AMPA) receptor trafficking and synaptic transmission [3]. Recent findings showed that Fmrp also affects other aspects of post-transcriptional gene regulation, including the stability of certain transcripts, activity-dependent mRNA transport, and RNA interference pathways [5]. Thus, Fmrp regulates the translation and trafficking of synaptic proteins, and subsequently affects synaptic plasticity and brain function.

The structure and sequence of the *fmr1* gene is conserved from invertebrates to mammals, allowing the development of various animal models to study the mechanism of the syndrome [6–10]. *Fmr1* knockout (KO) and conditional KO mice mimics the typical characteristics of FXS patients, including molecular, electrophysiological, neurological, and behavioral defects [7,8,11]. The imaging of KO mice brains revealed structural abnormalities of dendritic spines and changes in synaptic protein distribution that affect synaptic formation and plasticity [4,12]. In *Drosophila*, several *fmr1* mutants demonstrated an array of behavioral and developmental defects. As in the case of mammals, the morphology and connectivity of the synapses were altered [13]. In addition to the mouse and fly models, a zebrafish *fmr1* mutant (*fmr1*-/-) model for FXS was established [6]. The zebrafish is a transparent vertebrate that is suitable for genetic manipulations and live imaging of a simple and evolutionarily conserved CNS [14,15]. Consistent with the expression pattern of *fmr1* orthologs in mammals, *fmr1* expression in zebrafish is enriched in the brain [16]. Although an apparent phenotype was not observed in *fmr1*-/- larvae [6], adult *fmr1*-/- zebrafish demonstrated hyperlocomotor activity, impaired anxiety, and altered learning behavior [17,18]. Furthermore, reduced long-term potentiation and enhanced long-term depression was found in the telencephalon of adult *fmr1*-/- zebrafish, indicating deficient synaptic plasticity [18]. Thus, accumulating evidence points to a specific role of Fmrp in regulating synaptic proteins that mediate the structure and activity of neuronal circuits; however, the molecular mechanisms of this process remain unclear.

An intriguing mechanism that may be involved in FXS is adenosine-to-inosine (A-to-I) RNA editing. In drosophila, dFMR1 interacts and modulates the activity of adenosine deaminase acting on RNA (Adar) enzyme [13]. Adar acts on double-stranded pre-mRNA structures and deaminates A into I, which is recognized by the cell’s splicing and translational machineries as an equivalent to guanosine (‘G’), thus the A-to-G recoding process alters the mRNA coding sequences and enhances protein diversity [19]. In addition, RNA editing can affect alternative splicing [20] as well as RNA expression and stability [21], and Adar is a negative regulator of circular RNA (circRNA) formation [22]. Notably, deficient Adar can cause severe neurological defects or lethality in *Drosophila*, zebrafish, and mice [23–25]. Furthermore, key Adar target-sites are found in synaptic genes [26], and functional studies have shown that mild alterations in RNA editing levels in AMPA receptor subunits (GluRs) affect channel activity and synaptic plasticity [26,27]. However, although Adar-mediated RNA editing is linked to neurological alterations, the significance of this process in neurological disorders, such as FXS, and the global Fmrp-Adar mediated effect on the transcriptome, particularly neurological genes, are unclear. Here, the live imaging of neurites and synapses, as well as the video tracking of behavior, revealed abnormalities in neuronal circuit formation and locomotor activity in *fmr1*-/- larvae. Furthermore, thousands of clustered RNA editing sites were identified in the zebrafish transcriptome, and an interaction between Fmrp and Adar was determined. These findings, in addition to high-throughput microfluidic RNA editing quantification, suggest that RNA editing plays a role in the mechanisms that mediate neuronal circuit formation and behavior in FXS.

## Results

### Increased expression of the Fmrp target protein, mTor, is evolutionarily conserved in zebrafish

**Fig 1:**
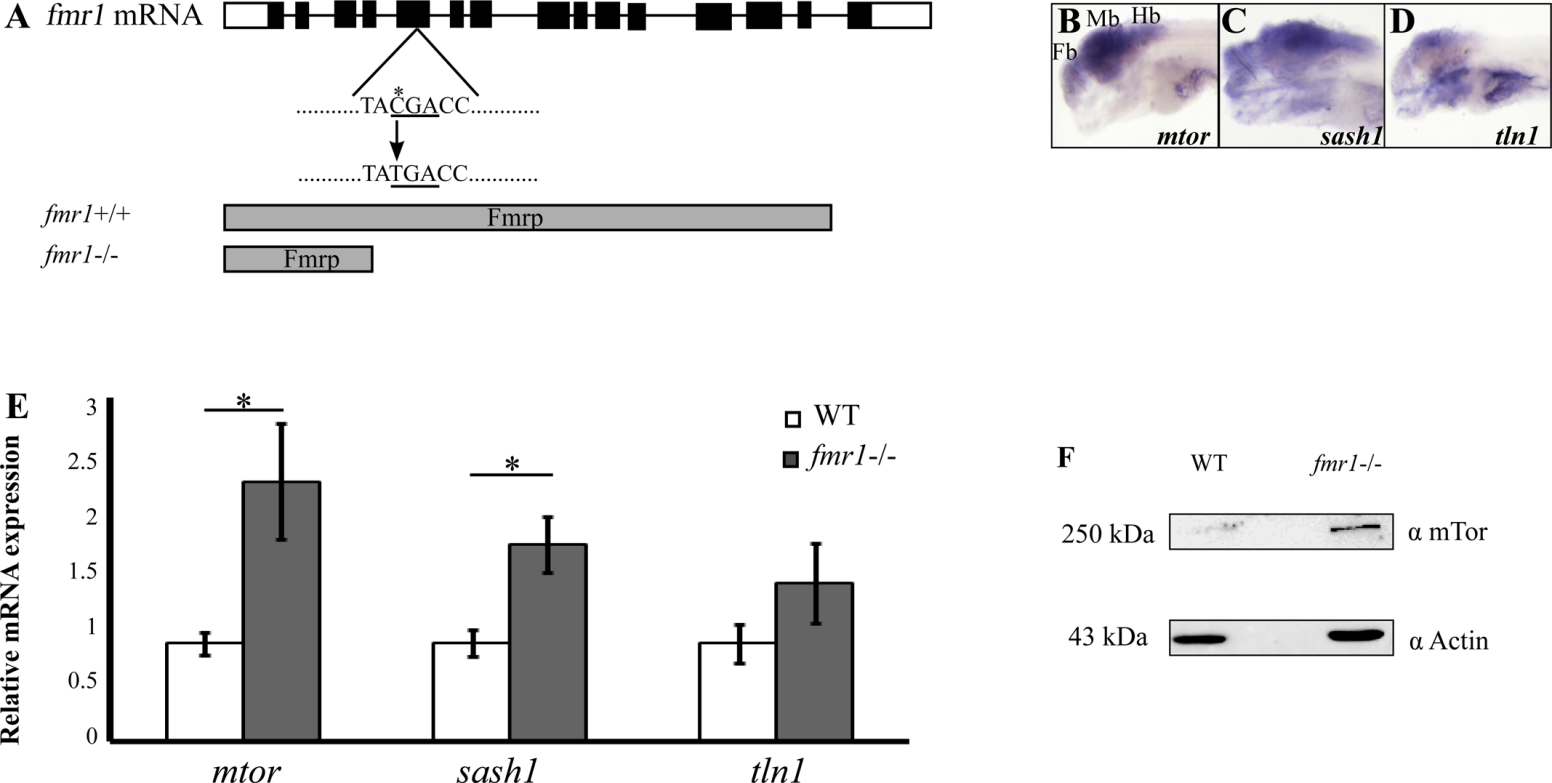
Increased expression of Fmrp-target genes in *fmr1*-/- zebrafish. **A.** The full mRNA sequence of the *fmr1* gene, including the CDS (black bars) and UTRs (white bars). A single C-to-T mutation at position 113 results in a premature stop codon and truncated protein (gray bars). **B-D.** Whole-mount ISH assays show the spatial expression of *mtor*, *sash1*, and *tln1* in 6 dpf WT larvae. Fb, forebrain; Mb, midbrain; Hb, hindbrain. **E.** Relative mRNA expression of *mtor*, *sash1*, and *tln1* in 6 dpf *fmr1*-/- *(*grey bars) and WT larvae (white bars). Values are represented as means ± SEM (*p<0.05, two-way *t*-test assuming unequal variances). **F.** Western blots show an approximate five-fold increase in the expression of mTor protein levels in *fmr1*-/- zebrafish brain tissue.

The RNA-binding protein Fmrp regulates the expression levels of a specific set of target proteins in humans [28]. Since an apparent morphological phenotype was not previously detected in *fmr1*-/- zebrafish larvae [6](Fig 1A), and in order to verify that the function of Fmrp in zebrafish is conserved with mammals, we sought to test the expression levels of three Fmrp target genes, *mtor*, *sash1*, and *talin1*, which showed elevated protein expression levels in FXS human brains [28]. mTor plays a key role in controlling protein homeostasis, cell survival, and synaptic density [29,30]. Sash1 is a member of the SLY family of signal adaptor proteins, and is essential in intracellular signal transduction [31]. Talin1 acts as an integrin-binding cytoplasmic adaptor that is a central organizer of focal adhesions [32]. To characterize the spatial expression pattern of *mtor*, *sash1*, and *talin1* in zebrafish larvae, whole-mount *in situ* hybridization (ISH) was used. Similar to *fmr1*, all three genes were widely expressed in the brain (Fig 1B–1D). In order to quantify the expression levels of these genes, quantitative reverse transcription polymerase chain reaction (qRT-PCR) was performed in 6 days post fertilization (dpf) *fmr1*-/- and wild-type (WT) larvae. The mRNA levels of *mtor* and *sash1* increased by approximately 2.5- and 2-fold, respectively, in *fmr1*-/- compared with WT larvae [*mtor* (WT = 1.03428, *fmr1*-/- = 2.73493), *p*<0.05; *sash1* (WT = 1.0617, *fmr1*-/- = 2.1338), *p*<0.05, Fig 1E]. These results show that loss of Fmrp results in elevated mRNA levels of target genes. Since *mtor* showed the highest increase in mRNA expression levels, we monitored its protein levels, specifically in the brain of *fmr1*-/- and WT zebrafish. Western blot analysis revealed an increase in mTor (250 kDa) protein levels in *fmr1*-/- compared with WT brains, while the protein levels of actin (43 kDa) were similar in both genotypes. Thus, similar to the case of mammals, the loss of Fmrp increases the expression levels of the mTor protein. Taking into account previous characterizations of physiological and behavioral deficiencies in *fmr1*-/- adult zebrafish [17,18], these results further establish the *fmr1*-/- larvae as a valid model for the study of the genetic and neurological mechanisms of FXS.

### Hyperlocomotor activity in *fmr1*-/- larvae

**Fig 2:**
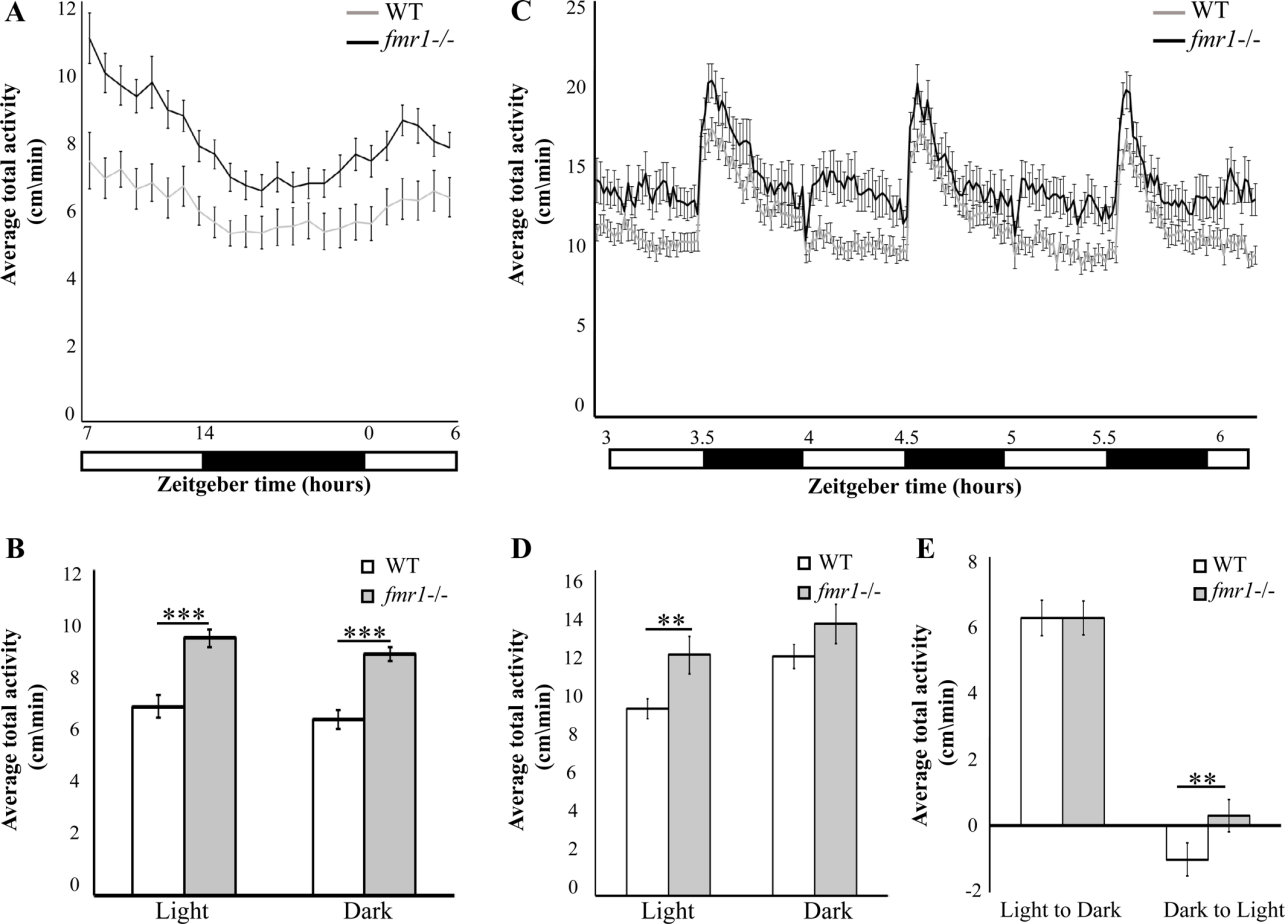
Hyperlocomotor activity and altered response to dark-to-light transition in *fmr1*-/- larvae. **A.** Locomotor activity (cm/min) recording was performed in 6 dpf *fmr1*-/- larvae (black line) and WT larvae (grey line) throughout a daily cycle under a 14 h light/10 h dark cycle. *fmr1*-/- larvae are hyperactive during both day and night (WT, n = 30; *fmr1*-/-, n = 34, ***p<0.0001). **B.** Average total activity (cm/min) during both day and night is presented for 6 dpf *fmr1*-/- and WT larvae. Values are represented as means ± SEM (*p<0.05, two-way *t*-test assuming unequal variances). **C.** Larvae were kept under alternating 30-min light/dark cycles during the day. The *fmr1*-/- larvae were hyperactive compared with WT larvae during the light periods (WT, n = 177; *fmr1*-/-, n = 179). **D.** Total average activity under alternating 30-min light/dark cycles during the day during both light and dark periods, is presented for 6 dpf *fmr1*-/- larvae and WT larvae. Values are represented as means ± SEM (*p<0.05, two-way *t*-test assuming unequal variances). **E.** Transition analysis demonstrating the differences in total average activity per genotype, calculated by comparing 5 min after and 5 min before light-to-dark and dark-to-light transitions. While the WT larvae showed reduced activity, the *fmr1*-/- larvae showed increased activity during the dark-to-light transitions (**p<0.005). Values are represented as means ± SEM. Statistical significance was determined by using a two-way *t*-test assuming unequal variances.

In the absence of Fmrp, FXS patients demonstrate hyperactivity, reduced anxiety-related behavior, and memory deficits [2]. Similarly, hyperlocomotor activity was observed in *fmr1* KO mice and *fmr1*-/- adult zebrafish [18]. To monitor locomotor activity in *fmr1*-/- larvae, high-throughput behavioral systems were used. Larvae were kept under light/dark conditions (LD, light 14 h: dark 10 h), and the rhythmic activity of 6 dpf *fmr1*-/- (*n* = 34) and WT (*n* = 30) larvae was monitored during the day and night. While both genotypes exhibited rhythmic locomotor activity that peaked during the day (Fig 2A), *fmr1*-/- larvae exhibited a 36% and 37% increase in locomotor activity during both day (WT = 6.961 cm/min, *fmr1*-/- = 9.483 cm/min, *p*<0.0001) and night (WT = 6.499 cm/min, *fmr1*-/- = 8.888 cm/min, *p*<0.0001), respectively (Fig 2B). These results indicate overall hyperlocomotor activity in *fmr1*-/- larvae, establishing that *fmr1*-/- larval behavior complies with the typically observed FXS phenotype. The response of *fmr1*-/- larvae to light and dark transition states was tested by exposing 6 dpf larvae to three cycles of alternating 30 min periods of light and dark during the day. The larvae responded to light and dark transitions with robust changes in locomotor activity (Fig 2C). Notably, during the light period, *fmr1*-/- larvae increased their locomotor activity by 28% compared with WT larvae (WT, n = 177, 10.410 cm/min; *fmr1*-/-, n = 179, 13.358 cm/min; *p*<0.005, Fig 2D), confirming that loss of Fmrp results in hyperlocomotor activity. Furthermore, behavioral analysis during the light-to-dark and dark-to-light transitions (comparing activity 5 min before and 5 min after the transition state) showed that while the response to dark stimuli was unaffected, the *fmr1*-/- larval response to light stimuli was adverse to that of the WT larvae. Notably, this tendency was repeated in all three light/dark cycles. Altogether, these results show hyperlocomotor activity and altered behavioral response to light stimuli in *fmr1*-/- larvae. These findings suggest that the loss of Fmrp affects locomotor activity, possibly due to the misregulation of synaptic proteins by Fmrp.

### Increased axon branching and synaptic density in *fmr1*-/- larvae

**Fig 3:**
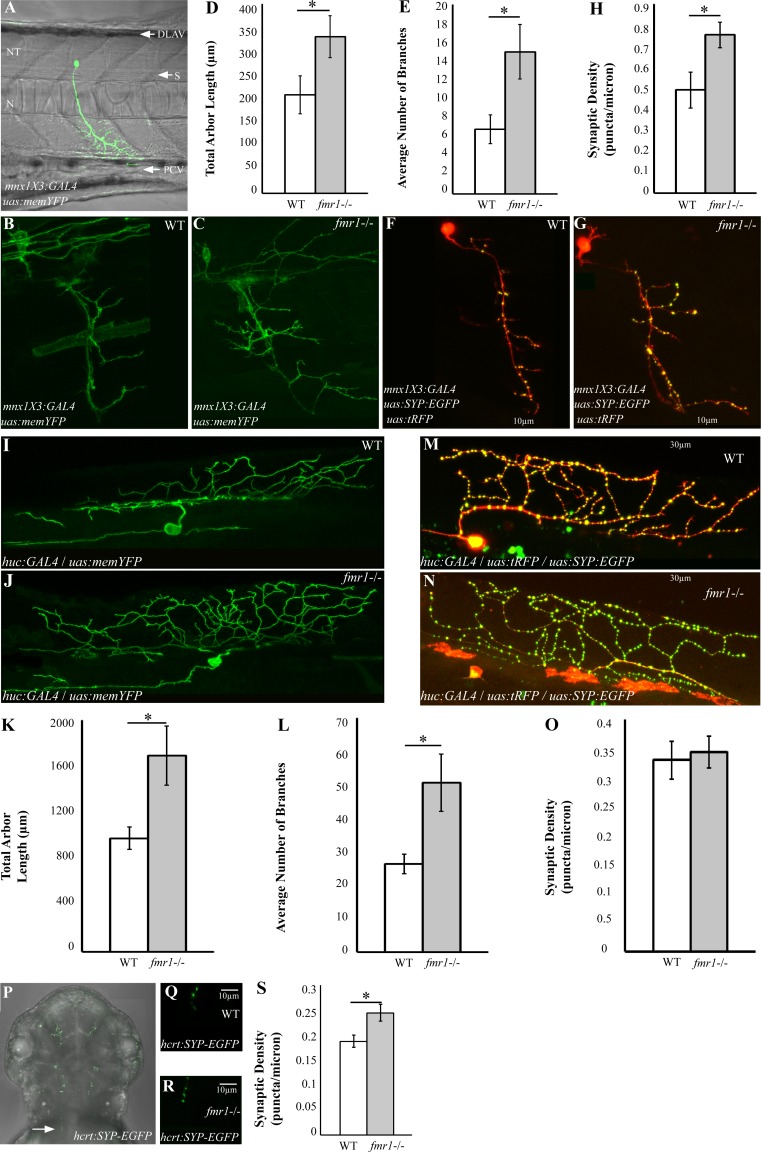
Increased synaptic density and axon branching in the CNS of *fmr1*-/- embryos. **A.** Wide-angled view of a confocal generated image of a single motor neuron in a 2 dpf embryo, which transiently expresses *mnx1X3*:*GAL4* and *uas*:*tRFP* constructs. DLAV, dorsal lateral anastomotic vessel; N, notochord; NT, neural tube; PVC, posterior cardinal vein; S, somite. **B-C.** Confocal imaging of motor neurons in 2 dpf *fmr1*-/- and WT embryos, which transiently express *mnx1X3*:*GAL4* and *uas*:*memYFP* constructs. **D-E.** Total arbor length **(D)** and number of branches **(E)** were measured in *fmr1*-/- (grey bars) and WT (white bars) embryos (WT, n = 17; *fmr1*-/-, n = 27, *p<0.*05*). Values are represented as means ± SEM. **F-G.** Confocal imaging of motor neurons in 2 dpf *fmr1*-/- and WT embryos, which transiently express *mnx1X3*:*GAL4*, *uas*:*SYP-EGFP* and *uas*:*tRFP* constructs. **H.** Total synaptic density was measured along the last 10 μm of a single branch of motor neurons in *fmr1*-/- and WT embryos (WT, n = 11; *fmr1*-/-, n = 17, *p<0.*05*). Scale bar = 10 μm. Values are represented as means ± SEM. **I-J.** Confocal imaging of spinal Rohon-Beard (RB) sensory neurons that project dorsally in 2 dpf *fmr1*-/- and WT embryos, which transiently express *huc*:*GAL4* and *uas*:*memYFP* constructs. **K-L.** The total arbor length (**K**) and number of branches (**L**) in the arbor of RB neurons were quantified in *fmr1*-/- and WT embryos (WT, n = 9; *fmr1*-/-, n = 10, *p<0.*05*). **M-N.** Confocal imaging of RB neurons in 2 dpf *fmr1*-/- and WT embryos, which transiently express *huc*:*GAL4*, *uas*: SYP-EGFP and *uas*:*tRFP* constructs. **O.** Total synaptic density was measured along the last 30 μm of a single branch of RB neurons in *fmr1*-/- (grey bars) and WT (white bars) embryos (WT, n = 10; *fmr1*-/-, n = 13). Scale bar = 30 μm. Values are represented as means ± SEM. Statistical significance was determined by two-sample *t*-test assuming unequal variances. **P.** Dorsal view of Hcrt neuron axons in a 2 dpf embryo, which transiently expresses the *hcrt*:*SYP-EGFP* construct. White arrow indicates the area analyzed. **Q-R.** Representative confocal imaging of Hcrt axons in 2 dpf *fmr1*-/- and WT embryos, which transiently express the *hcrt*:*SYP-EGFP* construct. **S.** Total synaptic density was measured along the last 10 μm of a single axonal branch of *hcrt* neurons in *fmr1*-/- and WT embryos (WT, n = 8; *fmr1*-/-, n = 9, *p<0.*05*). Scale bar = 10 μm. Values are represented as means ± SEM.

In the absence of an adequately functioning Fmrp, human FXS patients demonstrate severe cognitive and behavioral deficiencies. Taking into account these symptoms and the hyperlocomotor activity found in *fmr1*-/- larvae, we sought to resolve whether axon morphology and structural synaptic density of cholinergic motor neurons are affected in *fmr1*-/- live larvae. The *mnx1X3* enhancer [33] was used to fluorescently label motor neurons. The constructs *mnx1X3*:*GAL4* and *uas*:*memYFP* were co-injected into one-cell-stage *fmr1*-/- and WT embryos. At 2 dpf, *mnx1X3*:*GAL4*/*uas*:*mem*:*YFP* positive embryos were sorted out and single motor neurons were imaged (Fig 3A). Image analysis revealed that the total length of the axon arbors and the number of branches (Fig 3B and 3C) increased by 59% and 120%, respectively, in *fmr1*-/- compared with WT larvae (WT; n = 17, 208.050 μm, 6.823 branches; *fmr1*-/-; n = 27, 331.034 μm, 15 branches; *p*<0.05; Fig 3D and 3E). These results suggest that Fmrp acts to stabilize hyper-axon arborization in motor neurons. Since synaptogenesis guides the growth and branching of axonal arbors [34], the increased axon branching in *fmr1*-/- larvae could attest to a deficiency in the number and distribution of synapses. Therefore, the *mnx1X3*:*GAL4*, *uas*:*SYP-EGFP*, and *uas*:*tRFP* constructs were co-injected into *fmr1*-/- and WT embryos. At 2 dpf, synapse density was quantified by assessing the number of puncta along the axonal arbor of single motor neurons in both genotypes (Fig 3F and 3G). We found an increase of 53% (WT, n = 11, 0.490 puncta/micron; *fmr1*-/-, n = 17, 0.752 puncta/micron; *p*<0.05; Fig 3H) in synaptic density in *mnx1X3*:*GAL4*/*uas*:*tRFP/uas*:*SYP-EGFP*/*fmr1*-/- compared with *mnx1X3*:*GAL4*/*uas*:*tRFP/uas*:*SYP-EGFP*/WT embryos. These results show that loss of Fmrp increases total synaptic density in the axons of spinal motor neurons. Given that Fmrp is an inhibitor of synaptic protein translation [35], these findings suggest that Fmrp-dependent inhibition of synaptic proteins regulates axonal arborization and structural synaptic changes in cholinergic motor neurons.

The hyperlocomotor activity and disrupted behavioral response to dark-to-light transition states in *fmr1*-/- larvae, coupled with the broad expression of the *fmr1* gene, suggest that deficiencies in neuronal circuit formation are not restricted to motor neurons and may also be present in sensory neurons. In relatively early stages of zebrafish development, Rohon-Beard (RB) sensory neurons are the primary sensory spinal neurons [36]. They are located in the dorsal spinal cord and project axons toward broad areas in the periphery [37]. In order to test the role of Fmrp in RB axons, we imaged single RB neurons using the *huc* pan-neural promoter [38,39] in live embryos. The constructs *huc*:*GAL4* and *uas*:*memYFP* were transiently expressed in *fmr1*-/- and WT embryos and, at 2 dpf, positive embryos were sorted out and imaged (Fig 3I and 3J). We found that total arbor length and the number of branches increased by 73% and 92%, respectively, in *fmr1*-/- compared with WT embryos (WT, n = 9, 979.365 puncta/micron, 26.333 branches; *fmr1*-/-, n = 10, 1694.455 puncta/micron, 50.7 branches; *p*<0.05; Fig 3K and 3L). To quantify the number of synapses in live embryos, the *huc*:*GAL4*, *uas*:*SYP-EGFP*, and *uas*:*tRFP* constructs were co-injected into *fmr1*-/- and WT one-cell-stage embryos. At 2 dpf, synapse density was quantified in the axonal arbor of single RB neurons in both genotypes (WT, n = 10; *fmr1*-/-, n = 13; Fig 3M–3O). Imaging of synapses in these neurons revealed that synaptic density did not vary between *fmr1*-/- and WT larvae. These results show that Fmrp regulates axon branching in RB neurons that mediate sensory response.

Since Fmrp is widely expressed in the brain and spinal cord, and mGluR activation regulates Fmrp function, we tested whether loss of Fmrp affects structural synaptic density in glutamatergic neurons. We monitored synapse density in the glutamatergic hypocretin/orexin (Hcrt) neurons. These hypothalamic neurons innervate downstream glutamatergic nuclei, such as the locus coeruleus, and regulate feeding, reward, sleep, and wake [40]. The *hcrt* promotor was used to fluorescently label Hcrt axons [14]. The construct *hcrt*:*SYP-EGFP* was injected into one-cell-stage *fmr1*-/- and WT embryos. At 2 dpf, *hcrt*:*SYP-EGFP* positive embryos were sorted out and single axons, projecting dorsocaudally toward the spinal cord, were imaged (Fig 3P–3R). Image analysis of an *fmr1*-/- embryo revealed a 30% increase in synaptic density compared with a WT embryo (WT, n = 8, 0.1875 puncta/micron; *fmr1*-/-, n = 9, 0.2444 puncta/micron; *p*<0.05; Fig 3S). Altogether, these results show that loss of Fmrp increases synapse density along the axons of glutamatergic and cholinergic neurons in the brain and spinal cord.

### Fmrp-Adar interactions

**Fig 4:**
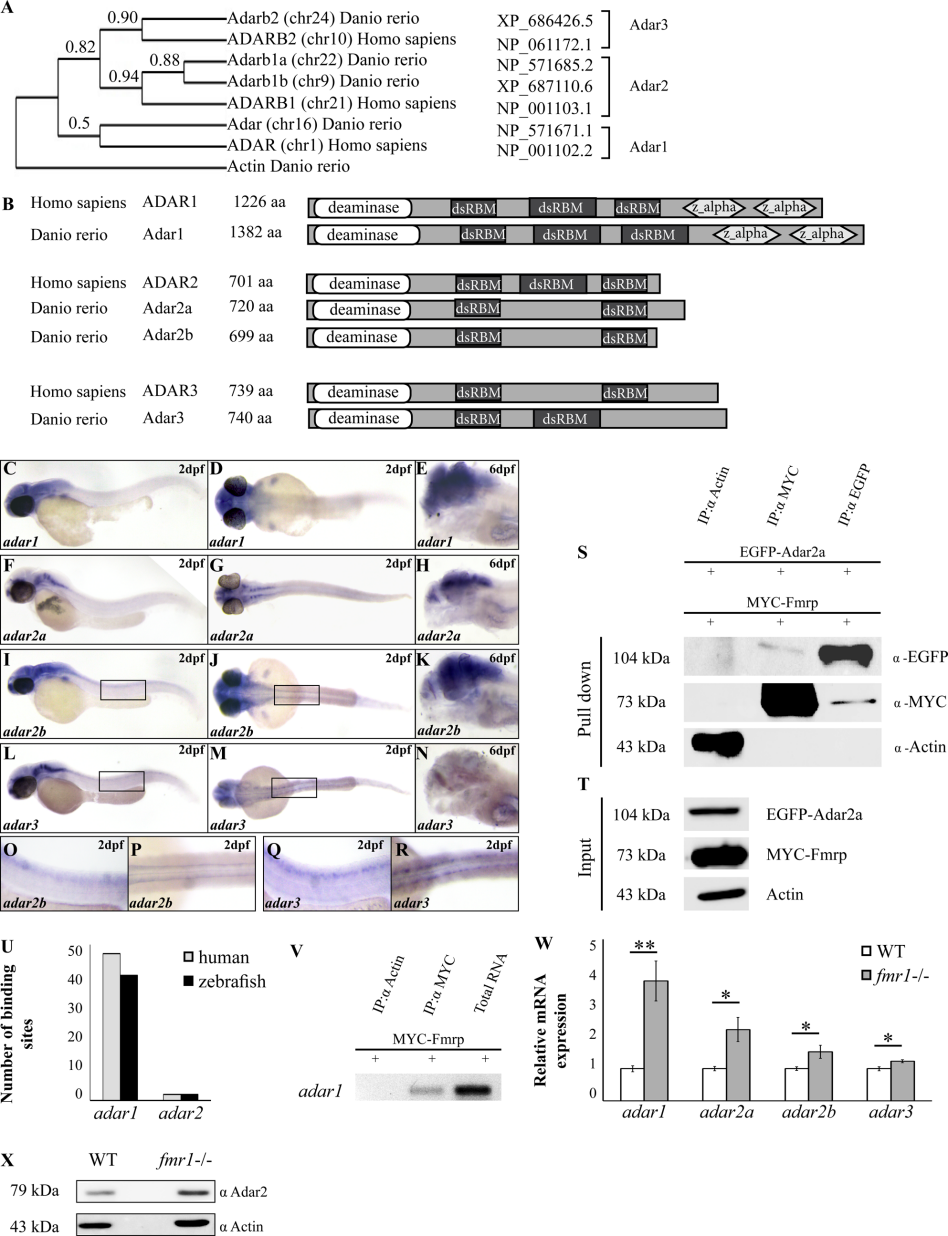
Fmrp-Adar interaction in zebrafish. **A.** Phylogenetic tree of zebrafish and human Adar proteins. Sequences are labeled with gene names, chromosomal locations, and accession numbers. To standardize and simplify the nomenclature, we named the genes Adar1-3, as indicated on the right side of each clade. Similarity values of each Adar member appear on top of each clade. **B.** Sequence conservation and motif distribution of Adar proteins in zebrafish and humans. Protein domains: adenosine deaminase domain (deaminase, white), double-stranded RNA binding motif (dsRBM, black) and zDNA binding domain (z_alpha, light grey). **C-R.** *In situ* hybridization showing lateral (**C**, **E**, **F**, **H**, **I**, **K**, **L**, **N**, **O**, **Q**) and dorsal (**D**, **G**, **J**, **M**, **P**, **R**) views of the spatial expression pattern of all four *adar* genes in 2 dpf **(C-D, F-G, I-J, L-M)** and 6 dpf **(E, H, K, N)** WT larvae. Expression is detected primarily in the nervous system. **O-R.** Selected regions (black frames in **L** and **M**) show *adar2b* (**O-P)** and *adar3* (**Q-R)** expression in the spinal cord of 2 dpf WT embryo. **S.** HEK-293T cells were transiently transfected with the zebrafish proteins Adar2a and Fmrp fused to EGFP and MYC, respectively (EGFP-Adar2a and MYC-Fmrp). Co-immunoprecipitation was used to detect Adar2a and Fmrp interaction. Actin was used as a negative control. The cell lysate was immunoprecipitated with anti-actin, anti-MYC, or anti-EGFP. Proteins were purified from the complexes and separated by SDS-PAGE. **T.** Western blot shows the protein content following the transfection prior to the immunoprecipitation. The proteins were detected with specific antibodies against MYC, EGFP, and actin. **U.** Computational sequence homology predicted the number of RNA recognition elements (RREs) in the CDS of *adar* genes that are recognized by Fmrp. **V.** RNA immunoprecipitation (RIP) assays show that Fmrp binds *adar1*. PCR amplification of *adar1* on RNA extracted from a RIP experiment conducted with anti-Actin and anti-MYC antibodies, and on total RNA extracted from HEK293T cells. **W.** RT-PCR assays showed that the mRNA expression levels of all four *adar* genes increased in 6 dpf *fmr1*-/- larvae (grey bars) when compared with WT larvae (white bars). Values are represented as means ± SEM. *p<0.05, **p<0.005, two-way *t*-test assuming unequal variances. **X.** Adar2 protein expression was analyzed by Western blot with specific antibodies against Adar2 and actin as a loading control. Elevated Adar2 protein levels of approximately 30% are present in *fmr1*-/- brains.

The mechanism by which the RNA binding protein Fmrp regulates axonal and synaptic structural changes in zebrafish is unclear. Recent findings on Drosophila showed that Fmrp modulates Adar enzyme activity [13], which, in turn, serves as a modulator of neuronal excitability and function as well as gene expression [26,41]. Since Adar-mediated A-to-I editing was shown to affect the function of synaptic proteins [26,42], we hypothesized that Adar expression and activity will be altered in fmr1-/- larvae. Initially, we sought to genomically characterize the zebrafish Adar family members. The Adar enzymes are highly conserved in metazoans, although the number of genes and isoforms varies between species [43]. Mammalian genomes encode three Adars: Adar and Adarb1 (Adar1 and Adar2, respectively) which are both catalytically active, and Adarb2 (Adar3), which is considered to be catalytically inactive [44]. An alternative promotor at the amino terminus of human Adar1 leads to the formation of two defined isoforms, commonly known as Adar1-p150 and Adar1-p110. While Adar1-p150 is localized in the nucleus and the cytoplasm, the shorter Adar1-p110 isoform is constitutively active and localized mainly in the nucleus [45]. Genomic analysis revealed that the zebrafish genome encodes four adar genes [43,46]. A phylogenetic reconstruction of the zebrafish and human Adar protein sequences revealed that zebrafish Adar proteins converge into three distinct clusters and that human Adar2 has two zebrafish orthologs (Fig 4A). Notably, all zebrafish Adar enzymes share common domain architecture consisting of a variable number of amino-terminal dsRBDs and a carboxy-terminal catalytic deaminase domain (Fig 4B). To determine the spatial expression of adar genes, we performed whole-mount ISH in WT zebrafish. At 2 dpf, all adars were primarily expressed in the brain and spinal cord, while at 6 dpf, adar transcripts were strongly expressed in the brain (Fig 4C–4R).

Although evidence of an interaction between Fmrp and Adar has been shown in *Drosophila* [13], it is not known if these proteins associate in vertebrates. To explore the biochemical interaction between Fmrp and Adar2a proteins, co-immunoprecipitation (Co-IP) was performed. HEK293T cells were transfected with constructs that expressed the zebrafish Adar2a and Fmrp proteins tagged with EGFP and MYC, respectively (Fig 4S). Extracts from HEK293T cells expressing EGFP-Adar2a and MYC-Fmrp (Fig 4T) were used for actin, MYC, and EGFP pulldowns, and eluates were subjected to SDS-PAGE electrophoresis (Fig 4S). We found that Fmrp was purified with Adar2a and vice versa. In contrast, actin did not pull down nor purify with either Fmrp or Adar2a (Fig 4S). These results indicate that zebrafish Fmrp and Adar2a proteins interact. Intriguingly, loss of Fmrp in FXS may affect the expression levels of Adar mRNA and protein. Previous work showed that FMRP can bind to many mRNA sequences via two major RNA recognition elements (RREs, conserved sequences: ACUK and WGGA) [28,35,47–49]. Specifically, 75 RRE sequences were mapped to *adar1* [47 in coding sequences (CDS) and 28 in 3’-UTR] and 4 sequences were mapped to *adar2* (2 in CDS and 2 in 3’-UTR). Using computational sequence homology, we searched for RREs in the CDS of zebrafish *adar1* and *adar2* genes. We found 42 sequences that exhibit 100% homology between the two species. Of them, 40 sequences were mapped to the CDS of *adar1*, and 2 sequences were mapped to the CDS of *adar2* (Fig 4U). Thus, we determined whether Fmrp binds to *adar1* mRNA using RNA immunoprecipitation assays. HEK293T cells were transfected with the zebrafish MYC-Fmrp, and anti-MYC as well as anti-Actin antibodies were used to pull down specific protein-mRNA complexes. Following total RNA extraction from the cells, cDNA was amplified and *adar1* was identified in cells precipitated with an anti-MYC antibody but not in cells precipitated with an anti-Actin antibody. These results show that Fmrp protein can bind *adar1* mRNA (Fig 4V). In order to quantify the effect of Fmrp on *adar* mRNA expression levels, a qRT-PCR was performed in 6 dpf *fmr1*-/- and WT larvae. All four *adar* genes showed increased expression levels in *fmr1*-/- larvae compared with WT larvae (Fig 4W). The mRNA levels of *adar1*, *adar2a*, *adar2b*, and *adar3* increased by 3.7, 2.2, 1.5, and 1.2 fold, respectively (*adar1*, WT = 0.834, *fmr1*-/- = 3.102, *p*<0.005; *adar2a*, WT = 1.03667, *fmr1*-/- = 2.2900, *p*<0.05; *adar2b*, WT = 0.9752, *fmr1*-/- = 1.48804, *p*<0.05; and *adar3*, WT = 1.0098, *fmr1*-/- = 1.24590, *p*<0.05, Fig 4W). Furthermore, Western blot analysis showed a 30% increase in Adar2 protein expression levels in *fmr1*-/- brains compared with WT brains (Fig 4X). These results show that Fmrp interacts with *adar1* mRNA and Adar2a protein, and that loss of Fmrp leads to increased expression of the *adar* genes and the Adar2 protein.

### Transcriptome sequence analysis uncovers a multitude of hyperedited (HE) RNA sites in zebrafish

**Fig 5:**
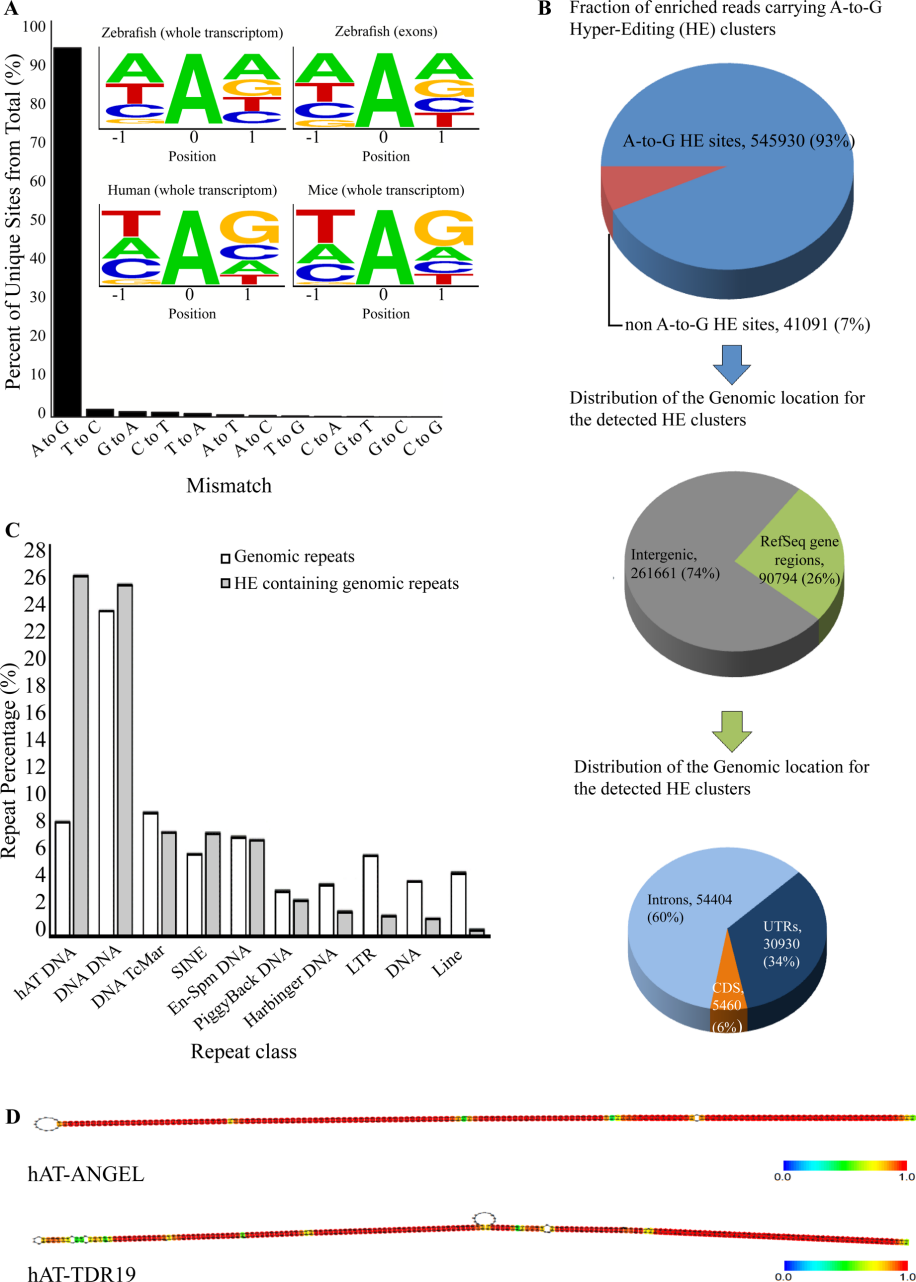
Transcriptome RNA hyperediting (HE) clusters in zebrafish. **A.** Out of the overall 12 possible mismatches between RNA and DNA, there was a cluster enrichment of A-to-G transitions (93%) compared with the other 11 mismatch possibilities (7%). Web-logo diagrams show the abundance of each nucleotide located 1 bp upstream (position -1) and 1 bp downstream (position +1) of every A/G mismatch found in zebrafish. Top panels show the results for both -1 and +1 positions in the zebrafish exons (top right) and whole transcriptome (top left). Consistent with the established motif, ‘G’ is the least represented nucleotide, with 8.4% in whole transcriptome and 11.4% in exons. Bottom panels show the results for both -1 and +1 positions surrounding the 42,500 editing sites comprising the RADAR dataset in humans (bottom left) and mice (bottom right). In position -1, similar to the case of zebrafish, ‘G’ is considerably under-representation with 8.4% and 4.4% in humans and mice, respectively. **B**. The distribution and number of A-to-I RNA hyperediting (HE) sites. The top chart represents all detected DNA-RNA mismatches. A-to-G mismatches are the majority (93%) of all mismatches. Middle and bottom charts show the genomic location of the detected HE clusters. **C.** Most of the detected RNA editing sites were found in repeats. Comparison between the distribution of the total and edited repeat families in the zebrafish genome showed an enrichment of the hAT family DNA repeats. While the hAT family occupies only 8% of total repeats in the entire zebrafish genome, it holds 26% of the total cluster containing sequences. **D.** *mfold* analysis of RNA secondary structure performed on the two most prominent DNA repeats (ANGEL and TDR19), which are members of the hAT family and account for over 11% of all sites detected. Structure analysis shows a long-stemmed dsRNA structure with palindrome traits that enable Adar binding and, consequently, RNA editing. Color code represents the strength of the nucleotide connection.

The location of known RNA editing sites in zebrafish is limited to a handful of sites [24,50,51]. To study whether RNA editing is altered in FXS, we initially assessed the global extent of RNA editing in zebrafish by analyzing transcriptome data. We probed RNA-seq datasets consisting of ~1.8Gb, strand-specific, 76-bp paired-end reads from a developmental study that contained 17 samples covering 8 different developmental stages [52] (GSE32898). RNA-seq data were analyzed by a bioinformatic pipeline aimed at detecting dense clusters of HE RNA sites [53]. This approach is most suitable for detecting editing in cases lacking genomic information derived from the same sample. A total of approximately 350,000 DNA-RNA mismatches were identified (S1 and S2 Tables). We calculated the prevalence of all possible 12 DNA nucleotide substitutions and found massive A-to-G mismatch enrichment (93%, Fig 5A, S1 Table) in intergenic regions, introns, UTRs, and CDS (Fig 5B). This large enrichment strongly suggests that these substitutions are the result of Adar activity. Next, we examined whether the nucleotide sequence context of the detected RNA editing sites complies with the sequence motif typical of Adar targets. Mammalian Adar1 and Adar2 enzymes have an apparent preference for RNA uracil (‘U’) at position -1 located 5’ to the editing target site, while guanine (‘G’) is the least constructive option [54]. Analyzing the sequence of adjacent zebrafish, HE sites revealed that, as in the case of mammals, ‘G’ is the underrepresented nucleotide at position -1 (Fig 5A). Since this position was shown to be imperative for RNA editing, our finding supports that the A-to-G mismatches are RNA editing sites. Further analysis of the genomic locations of the editing sites in humans and zebrafish revealed that the majority of the clustered sites (76%) are located in repeat sequences, mainly rich intergenic regions. Of these, we found that over 25% of all editing sites are located in the DNA hAT repeat family, which occupies about 8% of the zebrafish genome (Fig 5C). Using the *mfold* tool [55], we illustrated that both members of the hAT repeat family, ANGEL and TDR19, fold into stable dsRNA structures that are typical Adar substrates (Fig 5D). Altogether, these findings profile a genome-wide RNA editing map in zebrafish that includes ample RNA editing sites in hundreds of Adar target genes.

### Altered RNA editing levels within gene transcripts in *fmr1*-/- larvae

**Fig 6:**
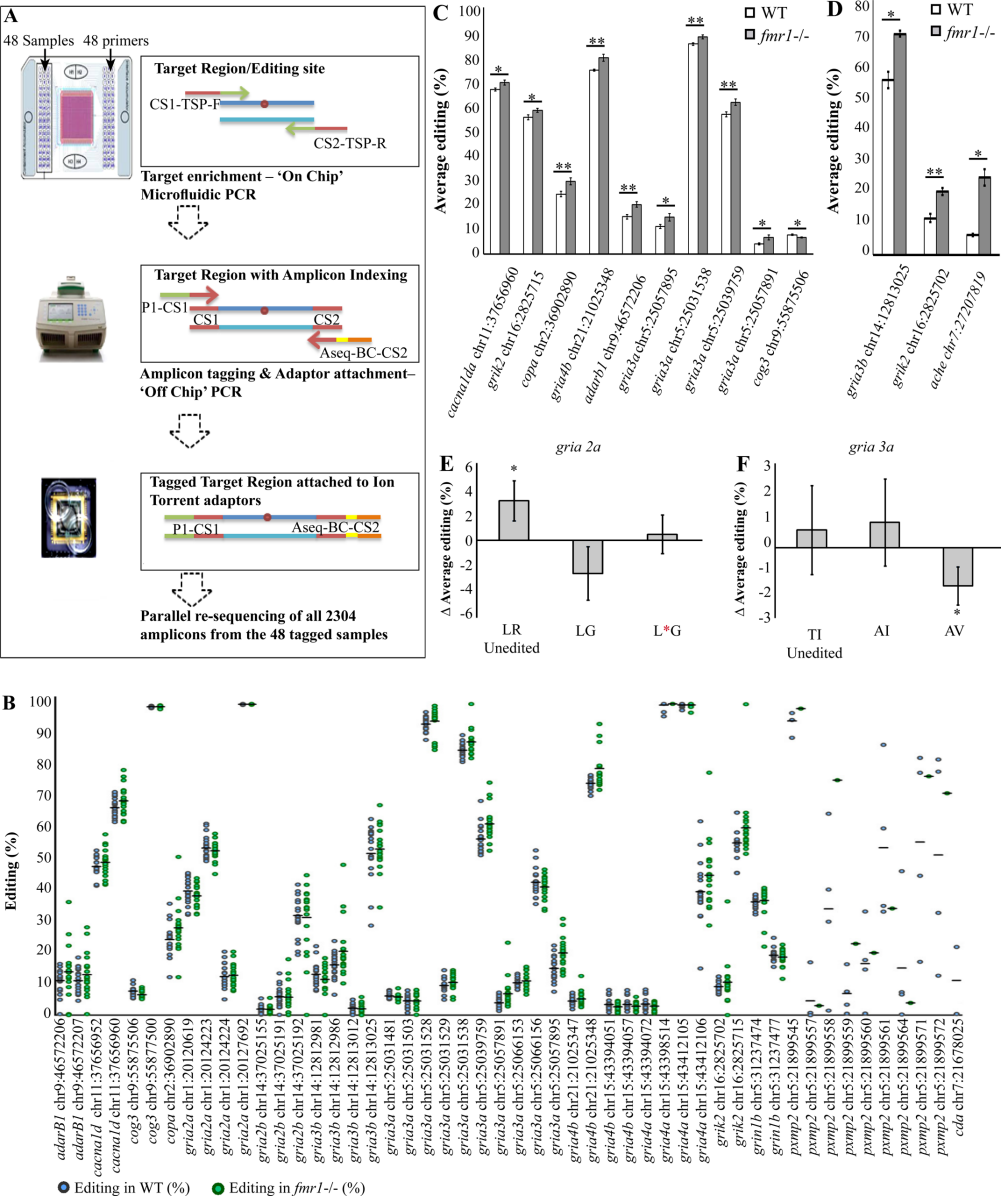
Targeted resequencing by mmPCR revealed differential RNA editing levels in *fmr1*-/- larvae. **A.** Schematic representation of the three major steps in the amplification and quantification of multiple RNA editing sites by next-generation sequencing. 1. A microfluidic-based PCR using the Fluidigm Access Array platform on the IFC chip (sample and primer inlets, black arrows) generates 48 targeted amplicons from 48 different samples. Schematic representation of the “on-chip” PCR; target regions (blue lines) contain a targeted RNA editing site (red circle) amplified by PCR with forward and reverse target-specific primers (TSP-F/TSP-R) fused to common sequences (CS1/CS2). 2. “Off-chip” PCR generates mini-library tagging, and the addition of IT-adaptor sequences creates 48 fully tagged and sequencer-compatible mini-libraries. Fusion primers containing CS1 and CS2 (red line primers) and the Ion Torrent PGM adaptor sequences P1 (green) and Aseq (orange) are used to generate completed amplicons (blue lines flanked by red lines). Barcode sequences (yellow) for sample indexing are fused to the Aseq-CS2 primer. 3. Parallel sequencing of the combined libraries on Ion Torrent-PGM. All mini-libraries are pooled together. **B.** Dot plot represents all calculated values of A/G ratios [percentages (dots) and means (black horizontal lines)] in the set of target sites that met all selection criteria in WT (blue circles) and *fmr1*-/- (green circles) RNA. **C.** The ten editing sites that exhibited significant differential RNA editing levels between *fmr1*-/- and WT larvae (*n* = 20 batches of 10 larvae for each genotype, *p<0.05, **p<0.005). **D.** Representative RNA editing sites showed increased editing levels in the brains of *fmr1*-/- zebrafish. *gria3b* showed a 14% increase, *grik2* showed an 8% increase and *ache* showed an 18% increase (*gria3b* and *grik2*: WT, *n* = 4; *fmr1*-/-, *n* = 5; *ache*, *n* = 3 per genotype, one brain per sample, *p<0.05, **p<0.005). **E-F.** Genes with multiple editing sites located in close proximity in the same amplified target region, were analyzed to quantify the relative abundance of all possible protein combinations formed by the editing pattern. Grey bars represent differences in the relative abundance of mRNA transcripts between WT and *fmr1*-/- larvae. **E.** In *gria2a*, LR (Leucine, Arginine) represents the genomically encoded unedited version that exhibited a 2.6% difference in relative abundance (*p<0.05). **F.** In *gria3a*, AV (Alanine, Valine) represents the double-edited form that exhibited a 1.6% difference in relative abundance (*p<0.05). Values are represented as means ± SEM. Statistical significance was determined by two-sample *t*-test assuming unequal variances.

The elevated levels of *adar* mRNA expression and Adar2 protein observed in *fmr1*-/- zebrafish (Fig 4), along with the identification of a multitude of clustered editing sites in the zebrafish transcriptome (Fig 5), provided the basis to examine whether the loss of Fmrp affects RNA editing levels in target genes. Until recently, in order to identify and quantify a single RNA editing site, traditional saturated PCR amplification of a single locus was used. The introduction of RNA-seq techniques led to the massive identification of new RNA editing sites; however, quantification of minute alterations in the levels of RNA editing is fairly limited due to typical low depth coverage of specific loci and the large dynamic range of RNA expression [56,57]. In this study, we focused on evolutionarily conserved RNA editing targets between mammals and zebrafish. We utilized a novel microfluidic-based multiplex PCR (mmPCR) approach to simultaneously amplify 48 target regions that contain the preselected RNA editing target sites across a 48-sample panel (Fig 6A, Materials and Methods) [58]. PCR products were index-tagged and subjected to deep sequencing, enabling single-molecule resolution of RNA editing levels in 6 dpf *fmr1*-/- and WT larvae (n = 10 batches of larvae per genotype). Before analyzing the A-to-G ratio per site, we ran a correlation test (S1 Fig). We found that the correlation between the number of reads taken from *fmr1*-/- and WT larvae is consistently high and does not affect editing levels (*p*<0.0001, two-tailed Pearson r = 0.978; S1 Fig). The zebrafish genome is highly polymorphic and does not consistently match the database (genome assembly Zv9, GCA_000002035.2); thus, for sequence comparison, we also sequenced and quantified genomic DNA from the same animals using the same targeted re-sequencing approach. This enabled a direct comparison of DNA and RNA sequences from the same source. In order to avoid quantification of genomic, single-nucleotide polymorphisms (SNPs) and sequencing errors, we set specific thresholds using the following criteria: i) sample incidence rate—target regions that were successfully captured in equal or more than 75% of the same genotype samples; ii) target site coverage—target regions with coverage depth of at least 400 reads; and iii) A/G ratio—an editing site with a ratio of [A/(A+G)] of at least 2%. Only target sites that met these criteria were further analyzed.

Out of the initial 70 predicted RNA editing sites included in our target set, 28 target sites met all criteria. An additional 35 novel and previously untargeted editing sites, located in close proximity to the targeted sites in the same genomic region, were also detected during data analysis. Of these, 24 sites were RNA editing sites that met the selection criteria (‘off-target’ sites, S3 Table). Finally, after applying all criteria, a cohort of 52 novel RNA editing sites was quantified and characterized in both *fmr1*-/- and WT larvae (S2 Table, Fig 6B). To validate the results, several RNA editing sites were sequenced using the customary Sanger method, and chromatogram analysis confirmed the presence of RNA editing sites (Materials and Methods, S2 Fig). Final analysis of RNA editing results showed *fmr1*-dependent differential RNA editing levels in ten sites (Fig 6C). These ten evolutionarily conserved RNA editing targets were located in seven genes. Four of these genes were synaptic genes: L-type calcium channel (*cacna1da*), ionotropic glutamate receptor kainate 2 (*grik2*), ionotropic glutamate receptor AMPA receptor subunit 4b (*gria4b*), and ionotropic glutamate receptor AMPA receptor subunit 3a (*gria3a*). Notably, *gria3a* showed differential RNA editing in four sites (Fig 6C, S4 Table). These results suggest that altered RNA editing, specifically in synaptic genes, might modulate synaptic structure and function in *fmr1*-/- larvae.

The changes in editing observed in whole *fmr1*-/- larvae were relatively mild. However, these changes may be larger in specific tissues. Therefore, we amplified and sequenced the genomic DNA (gDNA) and cDNA of three representative RNA editing sites, specifically from *fmr1*-/- and WT adult brains. Sequencing revealed that the difference in RNA editing levels between genotypes in *gria3b* and *grik2* genes, which showed no change when sampling whole larvae, increased to 14% and 8%, respectively. In addition, we quantified the levels of RNA editing in acetylcholinesterase (*ache*), which is a key enzyme in cholinergic synaptic transmission, and found an 18% increase in RNA editing levels in *fmr1*-/- compared with WT brains (Fig 6D). These results show Fmrp-dependent tissue-specific changes of RNA editing levels in brains and suggest that these relatively large changes may be present in other specific tissues.

Next, we employed a second analysis aimed to transform the quantification of site-specific editing levels into the complete formation of mRNA variants, which more accurately reflect the effect of RNA editing on transcriptome diversity. Analyzing all the editing sites located adjacently on a single molecule enabled us to determine the abundance of each mRNA sequence within the gene mRNA repertoire. This analysis showed that *gria2a* and *gria3a* contain multiple RNA editing sites on the same amplicon. When analyzed separately, insignificant, differential editing levels were found between *fmr1*-/- and WT larvae (S4 Table). However, when we analyzed the occurrence of RNA editing in conjunction with mRNA transcript formation, we identified small but significant changes in the relative abundance of the various mRNA transcripts generated by sequence recoding in *fmr1*-/- and WT larvae (S5 Table, Fig 6E and 6F). Analysis of the editing pattern of *gria2a*, which contains two adjacent editing sites (chr1:20124223 and chr1:20124224) showed a 2.6% increase in the genomically encoded, unedited form (LR) in WT compared with *fmr1*-/- larvae (S5 Table, Fig 6E: unedited form in WT = 43.2%∓1%, *fmr1*-/- *=* 40.6%∓0.9%, *p*<0.05). In *gria3a*, which also contains two adjacent editing sites (chr5:25066153 and chr5:25066156) the results exhibited a 1.6% decrease in the relative abundance of the fully edited version of AV formed by double editing in both T442A and I423V sites in WT compared with *fmr1*-/- larvae (S5 Table, Fig 6F; double edited form in WT = 9.8%∓0.4%, *fmr1*-/- *=* 11.3%%∓0.6%, *p*<0.05). This analysis shows that in clustered sites, even small changes in the editing levels can be manifested into different mRNA transcripts that can reshape synaptic proteins and thus contribute to functional diversity. Altogether, these results suggest that RNA editing sites, particularly in synaptic genes, are diversely regulated by Fmrp-mediated Adar activity. These molecular modifications may affect synaptic structure, axon processing, and the function of specific neuronal circuits that regulate behavior.

## Discussion

The epigenetic, neurological, and behavioral findings in the zebrafish FXS model suggest that Fmrp-mediated RNA editing plays a role in the molecular mechanisms that regulate structural plasticity of neuronal circuits that regulate behavior. In support of this model, Fmrp and Adar2a biochemically interacted, and loss of Fmrp increased the mRNA expression levels of the *adar* enzymes as well as Adar2 protein levels. Furthermore, we found that the zebrafish genome contains thousands of RNA editing sites, and that RNA editing levels are altered in conserved synaptic and neuronal transcripts in *fmr1*-/- zebrafish. For example, in accordance with the increase of RNA editing levels in glutamatergic and cholinergic genes, synaptic density was increased in the glutamatergic Hcrt neurons and the cholinergic spinal motor neurons in *fmr1*-/- zebrafish. Considering the role of these neurons in locomotor-activity regulation, the hyperlocomotor activity of *fmr1*-/- larvae may be linked to increased synaptic density in these neurons. Altogether, these results suggest an intricate interaction between Fmrp, Adars, and RNA editing in target genes that may affect the neurological symptoms of FXS patients. However, further research is needed in order to causally link RNA editing in specific targets with neuronal circuit-specific deficiencies in an animal model for FXS.

In order to understand the mechanism of FXS, we used *fmr1*-/- zebrafish. In humans [28], loss of Fmrp increased the expression levels of the inactive mTor protein. In mice, the levels of expression of the active form phospho-mTor increased, while the expression of the inactive form did not change [59]. As in the case of humans, in zebrafish, the loss of FMRP results in the increased expression of the mTor protein. The effect on phospho-mTor requires additional investigation. Taking into account the role of mTor in regulating structural, synaptic plasticity [30], it may play a role in the mechanism that regulates the assembly of neuronal circuits that regulate behavior in zebrafish and FXS. The behavior of *fmr1*-/- zebrafish was previously studied only in adult zebrafish [17,18]. However, at larval stages, the zebrafish model provides unique high-throughput and transparency advantages. Thus, we studied the role of *fmr1* in regulating neural circuit formation and behavior. High-throughput, video-tracking behavior systems were used to monitor the rhythmic locomotor activity during both day and night. As in the case of mammals and adult zebrafish [17,18], the larvae were hyperactive. Furthermore, under a 30-min alternating light and dark cycle, the larvae were hyperactive and their response to dark-to-light transition was altered. This hyperlocomotor activity is distinctive, since loss of gene function and neuron alterations typically result in reduced locomotor activity [60]. An intriguing explanation for the hyperactivity of *fmr1*-/- larvae could be that Fmrp mediates structural synaptic plasticity that affects behavior. A key role of Fmrp is inhibition of synaptic protein translation [61]. Thus, the loss of Fmrp may result in hyperactivity due to the augmented translation of synaptic proteins that interferes with synaptic degeneration and axon pruning processes. Supporting this hypothesis, the imaging of cellular and presynaptic fluorescent markers in live zebrafish showed increased axonal branching in both spinal motor and RB sensory neurons, and increased synaptic density in the axons of the Hcrt and motor neurons in *fmr1*-/- larvae. Since the spinal motor neurons regulate locomotor activity and the Hcrt neurons regulate arousal [40,62], the structural axonal and synaptic abnormalities found may induce the increased locomotor activity in *fmr*1-/- larvae. Furthermore, the abundance of immature dendritic spines is one of the neuroanatomical hallmarks of FXS in both humans and Fmr1-KO mice [12,63]. However, the role of Fmrp in regulating the development of axonal and presynaptic structures has yet to be fully characterized. In the cortex of FMR1-KO mice, the axonal arbors are diffused and demonstrate reduced connection probability and strength [64]. In *Drosophila*, Fmrp limits axon growth and facilitates activity-dependent pruning of axonal branches [65,66]. Thus, our findings in zebrafish support the findings in *Drosophila* and mice, and show that a lack of Fmrp results in excessive axonal processing and synaptic structures in neuronal circuits that induce locomotor activity.

The ability of a neuron to modulate synaptic protein composition and function relies on the fine-tuning of transcription and translation processes in both the nucleus and cytoplasm, as well as in cellular transport along the neurites. The RNA-binding protein Fmrp regulates translation and mRNA transport of synaptic genes. Another RNA-binding protein, the Adar enzyme, acts on an array of RNA molecules and expands protein diversity beyond that encoded by the genome. It also interferes with gene expression and coordinates miRNA biosynthesis to fine-tune neuronal plasticity and brain functions [19]. In *Drosophila*, Fmrp and Adar interact in the nucleus to regulate RNA editing. Furthermore, Adars act downstream to Fmrp to regulate synaptic morphology of the neuromuscular junction [13]. Similarly, we found that zebrafish Fmrp and Adar2a biochemically interact. Moreover, zebrafish Fmrp binds *adar1* mRNA. Notably, while in *Drosophila* Fmrp KO the levels of the single Adar protein [43] do not change [13], in *fmr1*-/- zebrafish, *adar* mRNAs and Adar2 protein expression levels are increased. These results suggest that Fmrp inhibits either Adar mRNA, protein or both, and further research is needed in order to elucidate the specific pathways. In either of the mechanisms, Fmrp likely affects Adar activity, which results in altered RNA editing levels in Adar-target genes. Furthermore, since a growing body of evidence links Adar activity to a broad array of the cell’s regulatory mechanisms, including the enhancement of A-to-I editing in the flanking intronic sequences of circRNAs [22], the potential increase of Adar activity in *fmr1*-/- larvae suggests a broad molecular modification of the transcriptome.

In order to quantify RNA editing in zebrafish, we first characterized a genome-wide profile of clustered RNA editing sites. The focus on clustered RNA editing sites allows an accurate evaluation of the prevalence of RNA editing even in the absence of genomic DNA data, and can be used as an indicator of overall Adar activity. Analysis of the zebrafish transcriptome showed that 0.2% of the total aligned reads were RNA editing sites (S1 Table), while the same analysis performed on RNA-seq data from the prefrontal cortex of healthy humans showed that 0.19% of the total reads were RNA editing sites [53]. Thus, as in the case of humans, these results demonstrate an extensive RNA editing process in zebrafish.

Whether the loss of Fmrp affects RNA editing in vertebrates was unknown. The Fmrp-Adar zebrafish protein interaction and the detection of hundreds of RNA editing sites within zebrafish genes provided the groundwork needed for RNA editing quantification in dozens of potential zebrafish target genes. We selected evolutionarily conserved Adar-targets in the CDS of annotated genes and used the mmPCR system to quantify RNA editing in those genes. The advantage of this system is its ability to simultaneously detect and accurately quantify A-to-G RNA editing events across multiple samples in a single experiment, independent of gene expression levels. We found Fmrp-dependent changes in RNA editing in several neuronal genes, particularly in glutamate receptors. Notably, although the RNA editing levels increased in most sites, the levels decreased in one specific site (cog3 chr9:55875506). This is also the case in the mutant *fmr1* fly, where the RNA editing levels increase in some genes and decrease in others [13]. The analysis of clustered RNA editing sites in *gria2a* showed that the overall prevalence of the mRNA containing the R/G recoding is higher in *fmr1*-/- larvae. Interestingly, this R/G editing site is located 2 nucleotides upstream to a splice site, which contributes to the generation of the Flip/Flop isoforms that modulate the kinetic properties of AMPA receptor channels, thus determining the time course of desensitization and re-sensitization [67]. Since the G forms of AMPA receptors have a faster recovery rate from desensitization compared to R forms [68], the increase in RNA editing at the R/G site may induce the synaptic response to glutamate. Thus, the changes in RNA editing in glutamate receptors may affect synaptic strength and morphology as well as cause hyperlocomotor activity in *fmr1*-/- larvae. Although the changes in editing observed in whole *fmr1*-/- larvae were mild, they were significant in several cases and might have been larger had the analyses been performed on a specific tissue or cell population. Indeed, quantification of RNA editing, specifically in the brain, showed greater changes in RNA editing levels. In addition, the relatively small changes in RNA editing were found in evolutionarily conserved targets that are carefully regulated. Furthermore, subtle editing changes are also typical in other model organisms for brain disorders. RNA-editing studies of various human neurological diseases, such as ALS, epilepsy, schizophrenia, and bipolar disorder, have all evidence for mild alterations in RNA editing [69–71]. Finally, since we found thousands of new RNA editing sites throughout the zebrafish genome, other untested target sites may exhibit more robust, Fmrp-dependent changes in RNA editing levels.

In summary, this study proposes a link between neurological deficiencies and RNA editing in the common mental disorder FXS. In order to elucidate the functional role of Fmrp in mediating Adar activity and RNA editing, further molecular and live-imaging studies should be performed. For example, establishing transgenic fish that overexpress edited and non-edited Fmrp-Adar target genes, combined with live imaging of the activity and structure of neurons and synapses throughout the brain, would help determine the pathogenesis of FXS symptoms. Taking into account that the zebrafish has become an attractive model for large-scale genetic and small-molecule screens, *fmr1*-/- larvae can provide the platform to elucidate the molecular mechanism and find therapeutic treatments for FXS.

## Materials and Methods

### Zebrafish husbandry

Adult zebrafish were reared and maintained in fully automated zebrafish housing systems (Aquazone, Israel; temperature 28±0.5°C, pH 7.0, conductivity 300 μS) under a 14-hour light/10-hour dark cycle, and fed twice a day. Embryos were generated by natural spawning and reared in water containing methylene blue (0.15%) in a 28±0.5°C, light-controlled incubator. All animal protocols were reviewed and approved by the Bar-Ilan University Bioethics Committee.

### DNA constructs, transient expression assays, and zebrafish lines

To prepare probes for whole-mount *in situ* hybridization (ISH) experiments, the full coding sequences of the following genes were amplified: mechanistic target of rapamycin (*mtor*, NM_001077211.2), SAM and SH3 domain containing 1a (*sash1a*, NM_001044819.1), talin 1 (*tln1*, NM_001009560.1), adenosine deaminase acting on RNA 1 (*adar1*, NM_131596.1), adenosine deaminase acting on RNA 2a (*adar2a*, NM_131610.3), adenosine deaminase acting on RNA 2b (*adar2b*, XM_682018.7), and adenosine deaminase acting on RNA 3 (*adar3*, XM_681334.5). All polymerase chain reaction (PCR) products were cloned into a pCRII-TOPO vector (Invitrogen, Carlsbad, CA), and served as a template to transcribe digoxigenin-labeled antisense mRNA probes. In order to generate the *pcmv*:*EGFP-Adar2a* and *pcmv*:*MYC-Fmrp* constructs, the CDS of *adar2a* and *fmrp* flanked with *SalI*/*KpnI* and *EcoRI*/*BglII* restriction sites, respectively, were amplified and double-digested with the appropriate enzymes. The *pcmv*:*EGFP* and *pcmv*:*MYC* vectors (kindly provided by Prof. Uri Nir, BIU, Israel) were double-digested with *SalI*/*KpnI* and *EcoRI*/*BglII*, respectively. The CDS of *adar2a* and *fmrp* were ligated into the digested *pcmv*:*EGFP* and *pcmv*:*MYC* vectors. The *pT2-uas*:*SYP-EGFP* construct was generated and used as described [60]. In order to generate the *mnx1X3*:*GAL4* construct, the *mnx1X3 promotor* located within the *p5E-mnx1(3X)* (kindly provided by Dr. Claire Wyart, ICM, France) was double-digested with *BamHI* and *HindIII*, and ligated into a *BamHI*/*HindIII*-digested *hcrt*:*GAL4* vector, replacing the *hcrt* promoter.

### Transient expression assays

To transiently express the following DNA constructs: *pT2-huc*:*Gal4-VP16*, *pT2-uas*:*tRFP*, *uas*:*memYFP*, *pT2-uas*:*SYP-EGFP* [60], and *mnx1X3*:*GAL4*, the constructs were diluted to a concentration of 40 ng/μl and microinjected, using a micromanipulator and a PV830 Pneumatic PicoPump (World Precision Instruments, Sarasota, FL), into one-cell-stage eggs. The embryos were kept in Petri dishes, and the pattern of EGFP expression was monitored throughout their development. The *fmr1*-/- line was kindly provided by Dr. Gordon X. Wang and Prof. Philippe Mourrian (Stanford University, CA). To minimize genetic variations, heterozygous (*fmr1*+/-) zebrafish were crossed and their progeny genotyped. Either *fmr1*-/- and its sibling WT adults or their progeny were used in each experiment.

### DNA and RNA extraction and cDNA preparation

DNA was extracted from larvae using the genomic DNA extraction kit (Invitrogen, Carlsbad, CA, USA) according to the protocol provided by the manufacturer. Total RNAs were extracted from the tissue with the Direct-zol RNA MiniPrep Kit (Zymo Research Corporation, Irvine, CA, USA) according to the procedure provided by the manufacturer. After DNase I treatment, 2–7.5 μg of total RNA was used to synthesize the first strand of cDNA with the iScript Advanced cDNA Synthesis Kit (Bio-Rad Laboratories Ltd., Berkeley, California, USA). cDNA was purified with the MinElute PCR Purification Kit (QIAGEN Sciences, Germantown, Maryland, USA) and concentrated using SpeedVac, if needed.

### Whole-mount ISH assay

In all ISH experiments, embryos and larvae were fixed in 4% paraformaldehyde overnight at 4°C, washed in phosphate-buffered saline (PBS), and stored in 100% methanol. The areas of mRNA expression were detected by ISH, as previously described [62]. Digoxigenin antisense riboprobes for *mtor*, *sash1*, *talin1*, *adar1*, *adar2a*, *adar2b*, and *adar3* were transcribed *in vitro* using the vector templates described above, and standard reagents followed the manufacturer’s instructions (Roche Applied Science, Nutley, NJ). ISHs were revealed using BM purple.

### Cell culture and transient transfection

HEK293T cell lines were grown in DMEM containing 10% heat-inactivated fetal bovine serum and 1% nonessential amino acids (Biological Industries, Beit Haemek, Israel), and incubated at 37°C under 5% CO_2_. HEK293T cells were transfected with 4 mg of *pcmv*:*EGFP-Adar2a* and *pcmv*:*MYC-Fmrp* vectors using the calcium phosphate method. The culture medium was changed 6 h after transfection, and cells were harvested 48 h later.

### Western blotting

Whole-cell proteins were extracted from mature fish brains or transfected HEK293T cell lines in lysis buffer containing 20 mM Tris, pH 7.5, 150 mM NaCl, 1 mM EDTA, 1% Nonidet P-40, 0.5% sodium deoxycholate, 2 mM Na_3_VO_4_, 1 mM NaF, and 10 mM b-glycerophosphate complement with protease inhibitors (cOmplete Protease Inhibitor Cocktail Tablets, Roche). Lysates were incubated for 30 min on ice, and the supernatant was collected after a 20-min spin at 14,000 rpm at 4°C. Protein concentration was determined by Bradford analysis (Bio-Rad Protein Assay Dye Reagent Concentrate, Bio-Rad, Hercules, CA, USA). A total of 30 mg protein extract was loaded per lane on 7.5% SDS polyacrylamide gel. After electrophoresis, proteins were transferred to a nitrocellulose membrane (BIO-RAD, Hercules, CA, USA), and the membrane was blocked for 1 h in phosphate-buffered solution [0.1% Tween (PBT) with 5% skim milk]. Next, the membrane was incubated in PBT with 5% skim milk containing the appropriate primary antibody: enhanced green fluorescent protein (EGFP) diluted 1:1000 sc-9996 (Santa Cruz Biotechnology, Dallas, TX, USA), anti-MYC diluted 1:1000 9E10 (Developmental Studies Hybridoma Bank, The University of Iowa), anti-mTOR dilution 1:750 GTX124771 (GeneTex, Inc., Hsinchu City, Taiwan), anti-Adar2 dilution 1:500 sc-393272 (Santa Cruz Biotechnology, Dallas, TX, USA, the antibody is expected to recognize zebrafish Adar2a and Adar2b alike], anti-actin dilution 1:500 sc-1616R (Santa Cruz Biotechnology, Dallas, TX, USA). After washing 3 × 5 min with PBT, the secondary antibody diluted 1:4000 [goat anti-mouse IgG-horseradish peroxidase: sc-2005, or goat anti-rabbit IgG-horseradish peroxidase: sc-2004 (Santa Cruz Biotechnology)] was incubated for 1 h in PBT with 5% skim milk. Membrane development was performed following 3 × 5 min washing with PBT using SuperSignal West Pico Chemiluminescent Substrate according to the manufacturer’s instructions (Thermo Fisher Scientific, Waltham, MA, USA). These Western blot experiments were performed twice on independent biological samples.

### Immunoprecipitation

Whole-cell proteins were extracted from transfected HEK293T cell lines as previously described (Western blotting). A total of 3000 μg protein extract was incubated overnight at 4°C with either EGFP diluted 1:100 sc-9996 (Santa Cruz Biotechnology, Dallas, TX, USA), anti-c-Myc Tag (9E10) Affinity Gel (BioLegend, San Diego, CA, USA), or b-actin 1:100 sc-1616R (Santa Cruz Biotechnology, Dallas, TX, USA). Antigen-antibody complexes were precipitated with protein A/G-Sepharose sc-2003 (Santa Cruz Biotechnology, Dallas, TX, USA) for 1 h at 4°C and washed 3 times with cold PBSX1. Precipitated proteins were then resolved by SDS-PAGE, blotted onto nitrocellulose membranes, and reacted with the appropriate antibodies: enhanced green fluorescent protein (EGFP) diluted 1:1000 sc-9996 (Santa Cruz Biotechnology, Dallas, TX, USA), anti-MYC diluted 1:1000 9E10 (Developmental Studies Hybridoma Bank, The University of Iowa), or anti-actin dilution 1:500 sc-1616R (Santa Cruz Biotechnology, Dallas, TX, USA).

### RNA immunoprecipitation

Whole-cell proteins and RNAs were extracted from Fmrp-MYC transfected HEK293T cell lines as described above. A total of 3000 μg protein-RNA extract was incubated overnight at 4°C with either anti-c-Myc Tag (9E10) Affinity Gel (BioLegend, San Diego, CA, USA), or b-actin 1:100 sc-1616R (Santa Cruz Biotechnology, Dallas, TX, USA). Antigen-antibody complexes of b-actin were precipitated with protein A/G-Sepharose sc-2003 (Santa Cruz Biotechnology, Dallas, TX, USA) for 1 h at 4°C. Antibody-protein-RNA complexes of both samples were washed 3 times with cold PBSX1. Coprecipitated RNAs were isolated by resuspending beads in 1 ml TRIzol reagent (Zymo Research, Irvine, CA, USA) followed by ethanol precipitation. cDNA was prepared as described above. PCR amplifications were performed using the following specific primers: *adar1*: 5'- CGGGCAATGCCTCGC -3' and 5'- AATGGATGGGTGTAGTATCCGC -3'.

### DNA sequencing and validation of A-to-G RNA editing results

DNA sequencing and confirmation of targeted re-sequencing data obtained via the mmPCR was performed in Hy-Labs (Rehovot, Israel) using standard sequencing methods. RNA editing sites were examined using Sequencher 4.10.1 Demo version and BioEdit version 5.0.6 (http://www.mbio.ncsu.edu/BioEdit/bioedit.html; File > Batch Export of Raw Sequence Trace Data). Editing sites were quantified by finding the maximal amplitude height of the A peaks (unedited) and the G peaks (edited), and also by calculating percentages of the population edited at each site [100% X (number of C nucleotides/total number of nucleotides)]. The University of California Santa Cruz (UCSC) genome browser was used to locate editing sites, mismatches between RNA and ESTs, as well as to establish conservation and homology between different species via alignments.

### Bioinformatic analysis of global RNA editing

The dataset of HE sites was created by analyzing deposited RNA-seq data [52] (SRA accession numbers SRR1028002, SRR1028003, and SRR1028004). Fastq files were aligned to the zebrafish genome (Zv9/DanRer7) using tophat, command: tophat -r 530 index fastq1, fastq1_replication fastq2, fastq2_replication fastq3, fastq3_replication. We then realigned the fastq files to the zebrafish reference genome, and added the splice junction file, achieved from the first run, as input. Command: tophat -r 530 -j splice_junctions_file; indexfastq1, fastq1_replication fastq2, fastq2_replication fastq3, fastq3_replication. Mpileup was then used to find differences between RNA sequences and the referenced-genome. Thresholds were set to collect sites that had more than five edited reads and that also had editing levels higher than 0.01 (as described, [53]).

### Analysis of clustered RNA editing sites

Several genes within our target set harbor a number of editing sites located in close proximity in the same amplified target region. In these cases, the calculated percentage of the A/G ratio did not reveal the actual impact of editing on the sequence composition of the mRNA transcripts leading off to the resultant protein. Since PCR amplification of target regions that contain more than one targeted editing site on the same PCR product (amplicon) was used, it enables identifying the presence of RNA editing sites and the correlations between neighboring editing sites.

### Compiling the target set of A-to-G editing sites

The Fluidigm access array (Fl-AA) system enables the simultaneous amplification of at least 48 different target regions across a 48-cDNA-sample panel on a single microfluidic device. Thus, 2,304 separate PCR reactions are performed simultaneously, followed by in-parallel next-generation sequencing, allowing the precise quantification of A/G ratios. The basic modus operandi of the (Fl-AA) system is in a singleplex PCR mode that limits the number of primer pairs used for the amplification of target regions to 48 per run. We employed a selection process to determine the 48 target regions. We designed the primer set to amplify evolutionarily conserved [72] editing targets that reside within the coding region of genes, preferably on editing sites that substitute for amino acids. The first step of the selection process was to screen the RADAR database of A-to-I RNA editing sites in humans and to select editing sites that are located within genes but that also reside outside of Alu repetitive regions [56,73]. Next, the UCSC and the lift-over tool were used to convert the list of all editing sites from its human genomic locations to zebrafish genomic coordinates. We compiled a list of 48 target regions containing a total of 70 novel zebrafish putative RNA editing sites located in 33 genes, of which 12 are directly linked with neuronal function. Of the total 70 target sites; 41 are located within the coding sequence of genes that mostly encode neuronal transcripts (59%), of which 27 target sites exert a non-synonymous effect (39%). Another 28 sites (40%) are located in the 3’-UTR of various genes, and one site was found to be located in an intron of the *adar2* gene [20]. Interestingly, this site is auto-edited by Adar2 and has been linked with the fine tuning of the mRNA re-coding process and affects behavior [43] (S3 Table). Finally, we compared the selected target-site list with the list of HE sites found in genes for shared entries, due to the fact that HE clusters occur with high probability in regions that generate dsRNA structures supportive of RNA editing. Indeed, we found that 7 of the 12 Refseq genes included in the final list of target regions were also found to contain HE clusters. This represents a 20-fold enrichment compared to the fraction of genes containing HE clusters in the entire zebrafish genome (2.9%).

### Amplifying targeted editing sites using microfluidic-based multiplex PCR (mmPCR) and next-generation sequencing

To distinguish between the *fmr1*-/- and WT samples, amplicons designed to contain the editing target sites were amplified across the sample panel containing both genotypes, using tagged fusion primers in a two-step consecutive PCR strategy. These primers were designed using Primer3.0 [http://frodo.wi.mit.edu/] and the 454 fusion primer design tool [http://eu.idtdna.com/scitools/applications/fusionprimers/default.aspx (IDT, Coralville, IA)]. Using the mmPCR method, we automatically assembled 2,304 unique PCR reactions, each reaction including a portion from each of the 48 samples screened with each one of the 48 primer pairs. The mmPCR amplification and tagging strategy is based on two consecutive PCR reactions, each performed with specific fusion PCR primers. The first PCR is performed "on chip" and generates amplicons of interest containing the target sites, which are flanked by designed common sequences [CS1 (fused to the forward primer)/CS2 (fused to the reverse primer)]. The second "off chip” PCR is performed on a thermal cycler and uses the first "on chip" PCR products as templates. The amplicons, now containing the CS regions conjoined (by the previous PCR), enable forming the attachment with sample-specific barcodes and Ion-Torrent PGM adaptors, thus making all 48 mini-libraries compatible for in-parallel NGS (Fig 6A).

Four μl of singleplex primer (4 μM per primer in 1X AA-loading buffer) was loaded into the primer inlets of the 48.48 Access Array IFC (Fluidigm, San Francisco, CA, USA). To prepare the cDNA templates, 2.25 μl of each cDNA sample was added to 2.75 μl of the presample mix containing the following enzyme and reagents from the Roche FastStart High Fidelity PCR System: 0.5 μl of 10X FastStart High Fidelity Reaction Buffer wo/Mg, 0.5 μl DMSO (5%), 0.1 μl 10 mM PCR Grade Nucleotide Mix (200 μM), 0.9 μl 25 mM MgCl_2_ (4.5 mM), 0.25 μl 20X Access Array Loading Reagent (Fluidigm, San Francisco, CA, USA), 0.05 μl of FastStart High Fidelity Enzyme Blend, and 0.7 μl of PCR grade water into the sample inlets of the 48.48 Access Array IFC (Fluidigm, San Francisco, CA, USA). After loading both samples and primers via IFC Controller AX (Fluidigm) script, the IFC was subjected to thermal cycling using FC1 Cycler (Fluidigm) with the following program for 40 cycles: 50°C for 2:00 min; 70°C for 20:00 min; and 95°C 10 min. For 10 cycles: 95°C for 15 sec; 59.5°C for 30 sec; and 72°C for 1 min. For 4 cycles: 95°C for 15 sec; 80°C for 30 sec; 59.5°C for 30 sec; and 72°C for 1 min. For 10 cycles: 95°C for 15 sec; 59.5°C for 30 sec; and 72°C for 1 min. For 4 cycles: 95°C for 15 sec; 80°C for 30 sec; 60°C for 30 sec; and 72°C for 1 min. For 8 cycles: 95°C for 15 sec; 59.5°C for 30 sec; and 72°C for 1 min. For 4 cycles: 95°C for 15 sec; 80°C for 30 sec; 60°C for 30 sec; and 72°C for 1 min; Finalizing with 72°C for 3 min.

Sample preparation included 1.0 μl of the 1:110-fold diluted PCR products as well as 15 μl of the presample mix containing the following enzyme and reagents from the Roche FastStart High Fidelity PCR System: 2 μl of 10X FastStart High Fidelity Reaction Buffer wo/Mg, 1 μl DMSO (5%), 0.4 μl 10 mM PCR Grade Nucleotide Mix (200 μM), 3.6 μl 25 mM MgCl_2_ (4.5 mM), 0.2 μl of FastStart High Fidelity Enzyme Blend, and 7.8 μl of PCR grade water. Four μl of primer mix from the 2 μM Access Array Barcode Library for Ion Torrent PGM Sequencer– 96 (P/N100-4911), utilizing the B-set; A–BC–CS2, and P1–CS1 barcode primer combination, was added to the sample mix. We used the following PCR program: 95°C for 10 min, 11 cycles of 95°C for 30 s, 60°C for 30 s, 72°C for 1 min, and 72°C for 5 min.

All 48-tagged mini-libraries were pooled into a single unified library and purified using the QIAquick PCR purification kit (QIAGEN Sciences, Maryland, USA). The output library was analyzed and quantified in the 2100 Agilent BioAnalyzer system using the HS DNA kit (Agilent Technologies, Santa Clara, CA, USA). After establishing the library dilution factor, the library underwent sequencing preparation using the Ion PGM Template OT2 200 kit, followed by the Ion PGM Sequencing 200-v2 kit, both according to the manufacturers' protocols. The fully processed library was loaded on the Ion 318 chip and sequenced using the Ion-Torrent PGM instructions (Life Technologies, Grand Island, NY 14072, USA).

### Data analysis of mmPCR results

We used FASTX Toolkit to demultiplex the raw reads. We used BWA26 to align the reads to a combination of the reference genome and exonic sequences surrounding known splicing junctions from gene models annotated in RefSeq and Gencode V12. We chose the length of the splicing junction regions to be slightly shorter than the reads in order to prevent redundant hits. For allelic-ratio count, we used the bases with a minimum quality score of 20. For read-depth count, we used the coverage of the representative sites in each amplicon. To obtain novel RNA-editing sites, we required variants to be supported by at least 10 mismatch reads with a base quality score and a mapping quality score ≥20. We also removed all known SNPs present in dbSNP (except SNPs of molecular type “cDNA”; database version 135; http://www.ncbi.nlm.nih.gov/SNP/), the 1000 Genomes Project or the University of Washington Exome Sequencing Project (http://evs.gs.washington.edu/EVS/). We used the Ion-Torrent PGM sequence output to detect and locate any A-to-G mismatches between the genomic DNA and the RNA sequences. Such mismatches were summed up and scored for their signal strength according to the fraction of ‘G’ reads of all ‘A+G’ reads [A/(A+G)*100].

### Real-Time PCR quantification assays

The relative mRNA quantification of *mtor*, *sash1*, *tln1*, *adar1*, *adar2a*, *adar2b*, and *adar3* was determined using qRT-PCR. Total RNA was extracted from 6 dpf embryos using the Direct-zol RNA MiniPrep kit (Zymo Research Corporation, Irvine, CA), according to the manufacturer’s instructions. For each tested gene, a total of five biological samples were used. Each biological sample contained a pool of 10 embryos. mRNA (1 μg) was reverse-transcribed using qScript cDNA SuperMix (Quanta BioSciences, Gaithersburg, MD), according to the manufacturer’s instructions. Relative transcript levels were determined by the 7900HT Fast Real-Time PCR System (Applied Biosystems, Foster City, CA). Triplicates of each cDNA sample were PCR-amplified using the PerfeCTa SYBR Green FastMix (Quanta BioSciences, Gaithersburg, MD) and the following specific primers:

*adar1*: 5'- ACCGCTGTGTTAAAGGAGAG -3' and 5'-AAAATAGTCTCATCGCCAGGG -3'; *adar2a*: 5'- CGGCAAGTACAAATCCAGGT -3' and 5'- CAGGTTGCGGTTTTCCTTTA -3'; *adar2b*: 5'- CTGGGAAGTCTGTATCATGCTG -3' and 5'- GTTGCCTTGCTTCTGTGTTAC -3'; *adar3*: 5'—GCCAGCTCGCTGTACTTCTC -3' and 5'- CAGGCACTCTTCAACTTCAGG -3'; *mtor*: 5'- CCCAGACTTATTCGCCCATAC -3' and 5'- CCATTTCCTCATCTCCAGTCC -3'; *sash1*: 5'- CATCTTCGGACAGTTTCTCCC -3' and 5'- GTACTCTTGTGCCAGGTCATC -3'; *tln1*: 5'- GTCAACACCATCACCAAACTG -3' and 5'- TTTAGCCACGTCCTTCACAG -3'.

The relative quantification of gene expression levels was normalized against β-actin: 5'- TGAATCCCAAAGCCAACAGAG -3' and 5'- CCAGAGTCCATCACAATACCAG -3' gene, and subjected to the ΔΔC_T_ method [74]. Gene levels were normalized by dividing the absolute levels of each sample with the average of all WT samples. Two-way *t*-test, assuming unequal variances, was used to compare the expression level of both genotypes. In all experiments, data were presented as means ± standard error of the mean (SEM).

### Behavioral assays

At 6 dpf, *fmr1*-/- larvae and WT were individually placed in 48-well plates under 14 h light/10 h dark cycles. Larva-containing plates were placed in the Noldus DanioVision tracking system (Noldus Information Technology, Wageningen, Netherlands) and acclimated for one hour prior to recording. Light intensity in the tracking system was 70LUX (25% in the operating software) for all experiments. To monitor rhythmic activity during a daily cycle, larvae were maintained under the same light-dark regime prior to the experiment. To monitor responses to light/dark transitions, larvae were subjected to 3 intervals of 30 min light/30 min darkness. Live video-tracking and analysis were conducted using the EthoVision XT 9 software (Noldus Information Technology, Wageningen, Netherlands) [62]. Four independent assays were performed, with a total of 177 and 179 larvae for each genotype in the light/dark-transition experiments, respectively, and a total of 30 and 34 larvae for each genotype in the daily-cycle experiment, respectively.

### Phylogenetic tree

Phylogenetic analysis was performed with PhyML 3.0 aLRT (http://www.phylogeny.fr/version2_cgi/one_task.cgi?task_type=phyml) using the "one click" program.

### Imaging and quantification

An epifluorescence stereomicroscope (Leica M165FC) was used to image fixed larvae. Pictures were taken using Leica Application Suite imaging software v. 3.7. For confocal imaging, embryos and larvae were placed in low-melting-point agarose (0.5–1.0%) on a specially designed dish filled with embryo water. A similar mounting protocol was used to image fixed embryos subjected to whole-mount ISH. Confocal imaging was performed using a Zeiss LSM710 upright confocal microscope (Zeiss, Oberkochen, Germany). To visualize single cells, optic sections of 0.5–1 micron were acquired. Positive cells were manually quantified using z-stack images of serial tissue slices. Images were processed using ImageJ (National Institutes of Health, Bethesda, MD) and Adobe Photoshop (San Jose, CA) software. Calculation of total arbor length and axonal branching in single motor neurons, RB sensory neurons, and Hcrt neurons was performed using NeuronJ plugin in ImageJ software (National Institutes of Health, Bethesda, MD). Synaptic density was calculated by quantifying the number of synapses per 10 μm in the axonal arbor of single motor neurons and Hcrt neurons, as well as 30 μm in RB neurons, using ImageJ software (National Institutes of Health, Bethesda, MD).

## Supporting Information

S1 Fig Uniformity in the mean number of aligned PCR products (reads) of all amplicons tested in *fmr1*-/- and WT samples. Correlation analysis of output reads shows independence of RNA sample origin. The high correlation between *fmr1*-/- and WT larvae reads shows unaffected coverage depth (Pearson correlation score, r = 0.978; R^2^ = 0.96).
(TIF)

S2 Fig Validation of RNA editing sites by comparing cDNA and genomic DNA sequences. **(A)** Validation of microfluidic-based multiplex PCR (mmPCR) results was performed by Sanger sequencing. Three representative RNA editing sites are shown. Sanger sequencing was performed on both genomic DNA (gDNA) and cDNA. Black arrow indicates the genomic location of each RNA editing site. **(B)** Comparison of RNA editing levels detected by mmPCR and Sanger sequencing in the representative target sites.
(TIF)

S1 Table Clusters of multiple editing sites (hyperediting) in zebrafish. The genomic location, strand, and sequence triplet of all HE sites detected.
(TXT)

S2 Table Clusters of multiple editing sites (hyperediting) in gene coding sequences in zebrafish. The genomic location, strand, and sequence triplet of all HE sites detected in the CDS region.
(XLSX)

S3 Table The target set of novel RNA editing sites. Locations, strand specificity, annotations, and mmPCR results of the target-set RNA editing sites.
(XLSX)

S4 Table Novel RNA editing sites in zebrafish. Gene name, genomic location, editing percentage, editing percentage SE, and annotation of novel RNA editing sites detected in *fmr1*-/- and WT larvae.
(TIF)

S5 Table RNA editing—cluster analysis. The levels of RNA editing recorded for each of the two adjacent editing sites in the *gria2a* and *gria3a* genes, as well as the calculated differential editing levels of these sites between *fmr1*-/- and WT larvae. The table also shows the calculated relative abundance for each mRNA transcript formed by editing in addition to the calculated differential abundance between *fmr1*-/- and WT larvae.
(TIF)
