# Supplementary figures and images for: Fmrp Interacts with Adar and Regulates RNA Editing, Synaptic Density and Locomotor Activity in Zebrafish
Source: PLoS Genet. 2015 Dec 4;11(12):e1005702. doi: 10.1371/journal.pgen.1005702 (PMC4670233; doi:10.1371/journal.pgen.1005702)

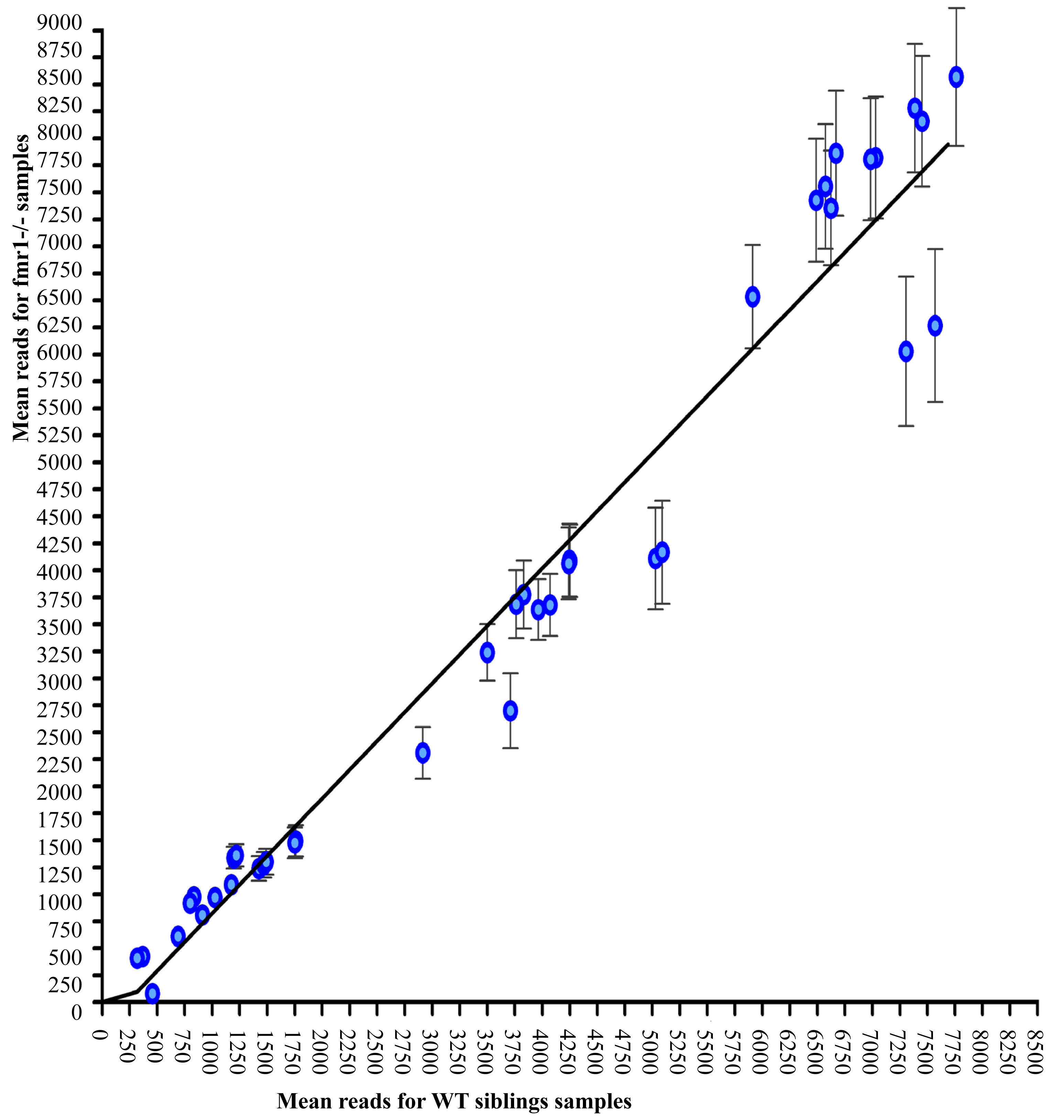

Supplement: S1 Fig — Correlation analysis of output reads shows independence of RNA sample origin. The high correlation between fmr1-/- and WT larvae reads shows unaffected coverage depth (Pearson correlation score, r = 0.978; R2 = 0.96). (TIF) [file pgen.1005702.s001.tif]

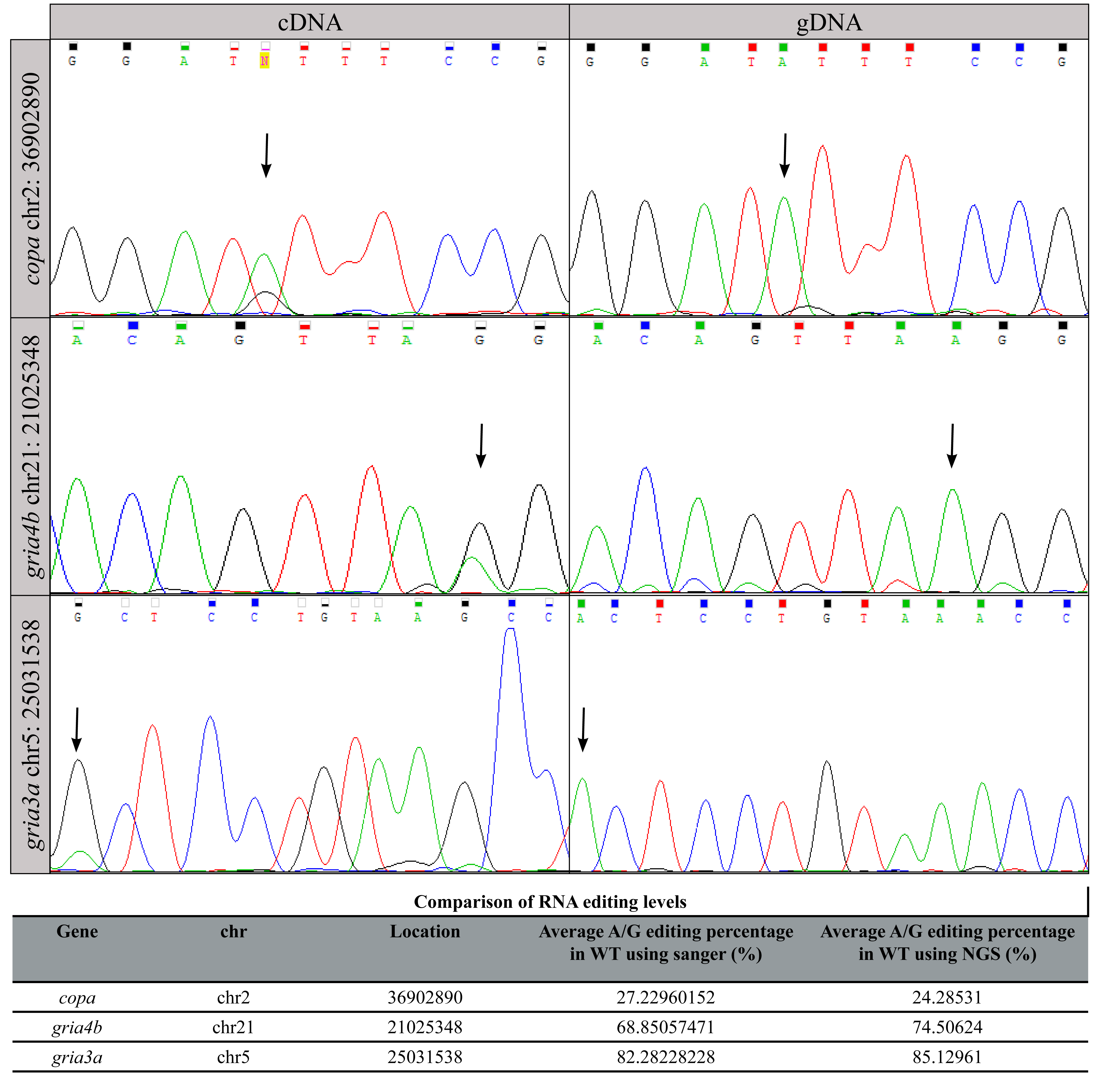

Supplement: S2 Fig — (A) Validation of microfluidic-based multiplex PCR (mmPCR) results was performed by Sanger sequencing. Three representative RNA editing sites are shown. Sanger sequencing was performed on both genomic DNA (gDNA) and cDNA. Black arrow indicates the genomic location of each RNA editing site. (B) Comparison of RNA editing levels detected by mmPCR and Sanger sequencing in the representative target sites. (TIF) [file pgen.1005702.s002.tif]

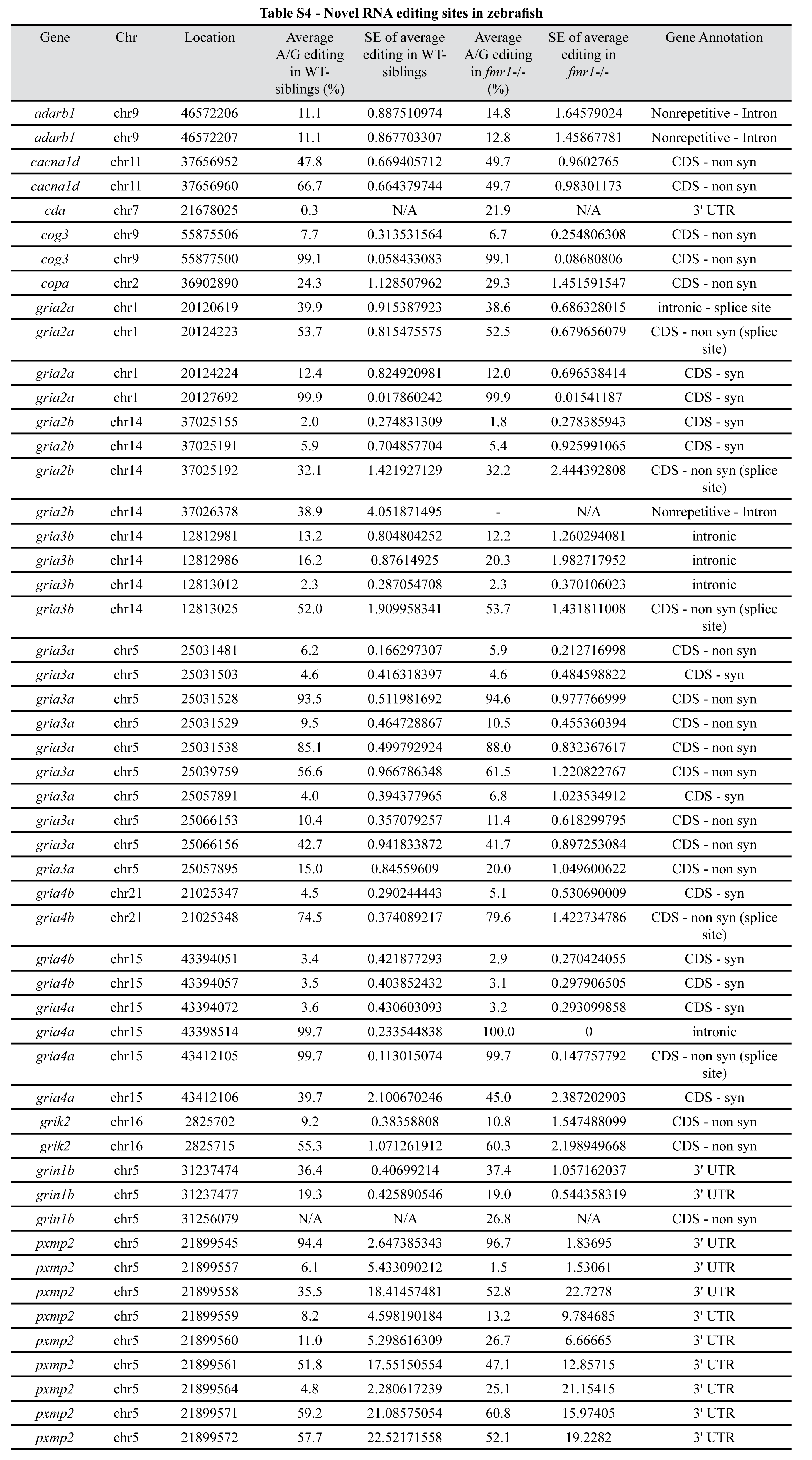

Supplement: S4 Table — Gene name, genomic location, editing percentage, editing percentage SE, and annotation of novel RNA editing sites detected in fmr1-/- and WT larvae. (TIF) [file pgen.1005702.s006.tif]

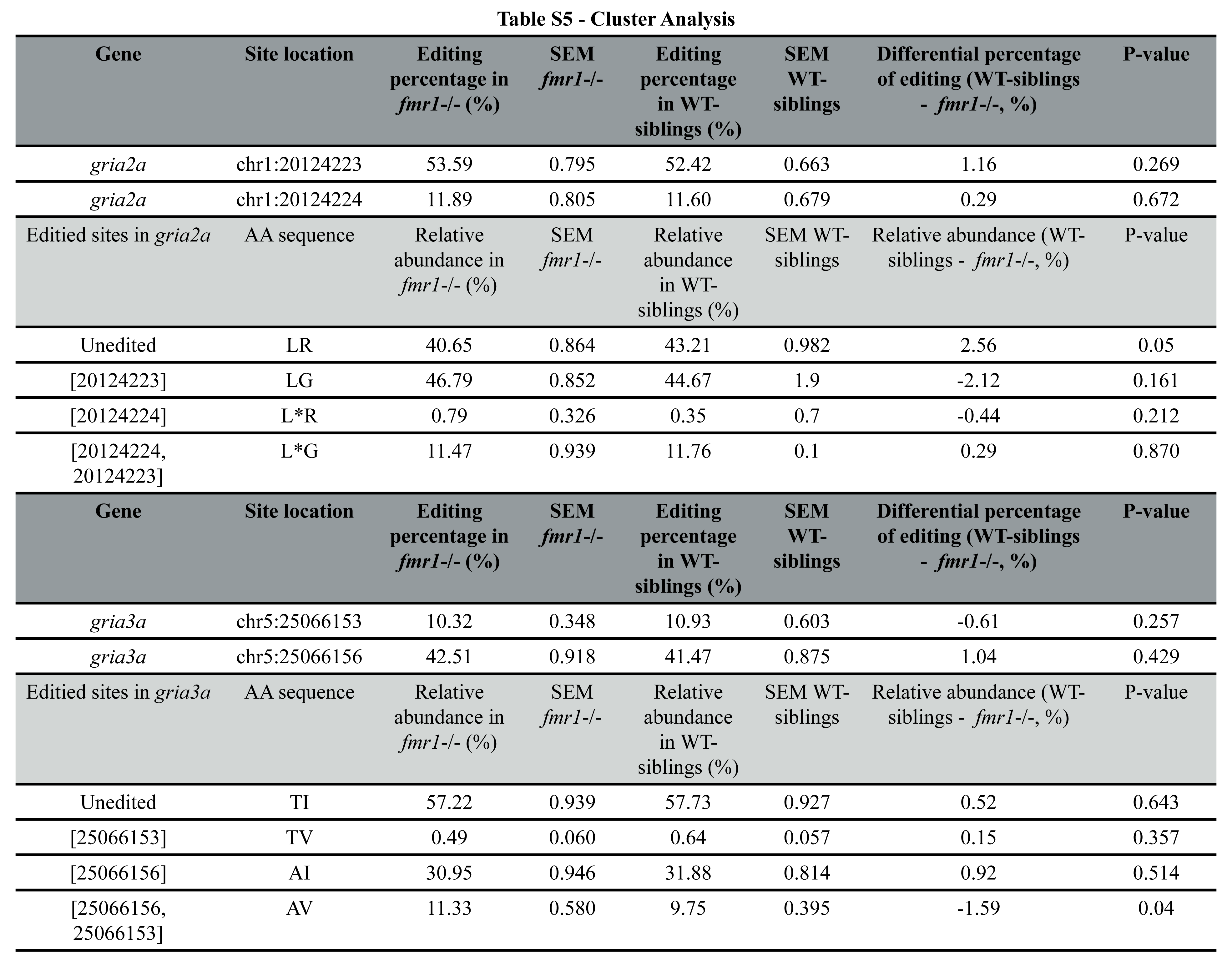

Supplement: S5 Table — The levels of RNA editing recorded for each of the two adjacent editing sites in the gria2a and gria3a genes, as well as the calculated differential editing levels of these sites between fmr1-/- and WT larvae. The table also shows the calculated relative abundance for each mRNA transcript formed by editing in addition to the calculated differential abundance between fmr1-/- and WT larvae. (TIF) [file pgen.1005702.s007.tif]
